# Potentials of Machine Learning in Predicting Key Features of Synthetic Antimicrobial Polymers

**DOI:** 10.1021/acspolymersau.5c00140

**Published:** 2026-04-27

**Authors:** Lena Dalal, Deborah Barker, Nicholas J. Warren, Olivier J. Cayre, Sebastien Perrier

**Affiliations:** † Department of Chemistry, University of Warwick, Gibbet Hill Road, Coventry CV4 7AL, U.K.; ‡ Department of Chemical and Process Engineering, 4468 University of Leeds, Woodhouse, Leeds LS2 9JT, U.K.; § Warwick Medical School, University of Warwick, Coventry CV4 7AL, U.K.; ∥ Faculty of Pharmacy and Pharmaceutical Sciences, Monash University, 381 Royal Parade, Parkville, Victoria 3052, Australia

**Keywords:** antimicrobial polymers, RAFT, machine learning, random forest, gradient boosting

## Abstract

As the global rise in antimicrobial resistance calls for new therapeutic strategies, synthetic antimicrobial polymers (SAMPs) have emerged as promising alternatives to host-defense peptides, offering tunable structures and reduced limitations. In this work, we employed machine learning (ML) approaches to elucidate the structure–activity relationships of a library of polyacrylamides systematically varied in (1) amine side-chain chemistry, (2) chain length, (3) cationic amine ratio, and (4) polymer architecture. The library consisted of 23 different polymer designs, 3 of which exhibited low minimum inhibitory concentrations (MIC) against different bacterial strains, and 5 of which caused low red blood cells agglutination. Among the evaluated ML algorithms, regression random forest and gradient boosting regression consistently reproduced feature importance and maintained stable decision-tree structures, with gradient boosting outperforming random forest in predictive power. Gradient boosting achieved RSME values of 20, 6, 13 and 12 μg/ml, respectively, for each modelled MIC of 4 bacterial strains: *Pseudomonas aeruginosa* PA14, *Pseudomonas aeruginosa* LESB58, *Staphylococcus aureus* USA300 and *Staphylococcus aureus* Newman (total data range 64-513 μg/ml). RSME for modelled hemagglutination was 1 μg/ml. Calculation of feature importances and visualisation with beeswarm and waterfall plots highlighted the contribution of individual polymer features through Shapley additive explanations (SHAP). All bacteria strains considered, the type and percentage of cationic monomer are the most important features determining best-performing SAMP designs. Collectively, our findings demonstrate that boosting-ensemble methods offer consistent robust predictive capability and can serve as effective tools for forecasting the potency and toxicity of future SAMP designs, with potential for application in larger, multi-sourced libraries.

## Graphical abstract

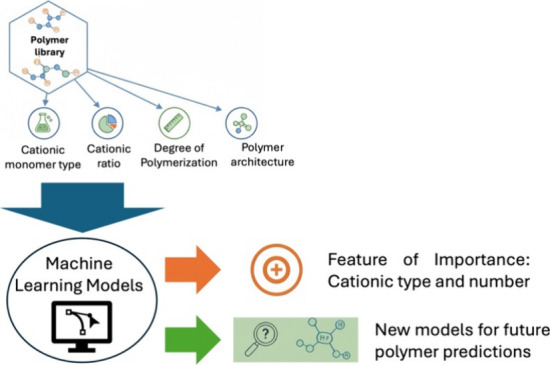

## Introduction

Antimicrobial resistance has become a significant global concern,^1^ with an urgent need to develop new generations of antibiotics.^2^ Among potential therapies, host-defense peptides (HDPs), generated by the human body as a first line of defense against infection, have been highlighted as promising broad-spectrum antibiotics.^3^ HDPs are short peptides consisting of a combination of less than 50 amino acids in secondary structure conformation, and this amino acid combination is crucial to their antimicrobial and immune response, as well as their selectivity toward microbial cells.^4^ HDPs comprise of a mixture of hydrophobic and cationic amino acids distributed to yield a net positive charge at physiological pH.^5^ This unique amphiphilic structure enables them to be soluble in the physiological aqueous environment while maintaining their ability to partition within the phospholipid microbial membrane.^6^ Moreover, the dominant cationic charge of HDPs prompts electrostatic interactions with the microbial cell membrane, dominated by negatively charged lipids. These interactions lead to the disruption of the membrane structure, formation of pores, and eventually apoptosis.^7,8^ However, the mammalian cell membrane consists of zwitterionic phospholipids (i.e., phosphatidylglycerol, cardiolipin) oriented outward, which minimizes interactions with HDPs, thus promoting the peptides’ selectivity toward microbial cells.^9–11^

This has prompted an extensive search for synthetic antimicrobial polymers (SAMPs) that replicate the activity of HDPs, while offering enhanced stability and a tunable chemical structure. Among the most versatile SAMPs reported to date are polymers functionalized with amino-acid-mimicking groups,^12,13^ built on diverse backbones including, but not limited to, polynorbornene,^14^ polyethylenimines,^15^ polynylon-3,^16–18^ polyvinylpyrrolidones,^19^ poly­(2-oxazoline)­s,^20,21^ polyurea,^22^ methacrylates,^23–27^ and acrylamides.^27–29^

Many structural parameters were found to affect the potency of SAMPs, in particular molecular weight, polymer backbone structure, type of cationic moieties, and its ratio to the hydrophobic monomer of choice, as described by Hartlieb et al.,^30^ Pham et al.,^31^ and Locock et al.,^24,32^ who focused mainly on copolymers of methacrylate and acrylamides derivatives. Well-defined antimicrobial polymethacrylates or polyacrylamides can be obtained via controlled radical polymerization techniques such as copper(0)-mediated radical polymerization^33^ and reversible addition–fragmentation chain-transfer (RAFT) polymerization.^34–36^ RAFT polymerization not only provides a good control over the polymers molecular weight but also allows for telescoping synthesis of multiblock copolymers with narrow dispersity.^37^

In a previous work by Perrier and co-workers^38–42^ polyacrylamides selected to resemble the amino acid functional groups found in innate HDPs (e.g., lysin, arginine, and isoleucine) showed great potential for future topical antimicrobials mimicking HDPs with improved resilience. These monomers’ high propagation rates (*k*_p_) facilitate their rapid and controlled polymerization via RAFT polymerization, allowing for the control of the cationic moiety distribution along the polymeric chain, to achieve the desired amphiphilic balance of HDPs with higher stability against degradation.^43^ A recent study^44^ showed that the main factors influencing the therapeutic profile of acrylamide-derived SAMPs include the type of cationic moieties and their ratio, the polymer chain length, and sequence. However, elucidating the structure–activity relationship (SAR) and optimizing the design of antimicrobial polymers is a time-consuming process, which calls for the utilization of automated methods that can capture highly non-linear behaviour in the data, handle several design features simultaneously, and reduce bias inherent in manual SAR interpretations.^24,30–32^

Recent advances in artificial intelligence tools provide a remarkable path toward more efficient and robust SAR analysis and material design. Particularly, machine learning (ML), a subset of AI that involves algorithms designed to generate predicted outcomes from historic data, appears to be a powerful tool to guide the design of the next generation of antimicrobials.^45,46^ In this approach, in silico screening minimizes the load of the experimental work required, as it can narrow down the number of potential candidates by predicting activity patterns.^47^ It can also foresee hidden patterns when screening the database for drug candidates, e.g., antibiotics, that otherwise might be missed as prospective candidates.^48^ The application of ML in the high-throughput screening of antibiotic candidate libraries has been reported recently,^49–53^ but a number of challenges are still to be addressed.^54^ The typical workflow for ML includes data collection, feature engineering, model selection and validation, and finally model application.^55^ Data can be sourced from direct experimental work, published literature, or existing databases. Experimental data have the advantage of controlled parameters, particularly for polymers, which ensure consistency and reliability. However, it is time-consuming and typically yields smaller data sets. In contrast, utilizing published research provides access to extensive data sets within a shorter time frame, but the diversity of experimental methods and data reporting standards may impact the quality and accuracy of ML predictions.^55,56^ Moreover, the accuracy of predictions relies on identifying relevant input variables (that is, design features) that influence the output performance. Hence, it is critical to consider the relevant features or descriptors to be utilized in the ML pipeline in an early stage, based on experimental knowledge but preferably before extensive data collection. This raised a challenge for HDPs when screening through their wide range of structures and sequences,^54,57^ and similarly, it could also be limiting for the use of ML models for SAMPs.

Another challenge is selecting an appropriate ML model, as identifying the optimum model for the data set is critical for successful predictions. There are many ML techniques to choose from for medicinal chemistry, i.e., decision tree, random forest (RF), gradient boosting (GB), and artificial neural networks, including algorithms for both regression and classification methods.^48^ Model selection considers the nature of the data set itself, computational time restrictions, and additional insights that may be available for each specific model. One insight that can be obtained from ML models is feature importance (FI), which gives the contribution of each feature to the model output and can facilitate the identification of SAR. Permutation FI can be performed after model selection and fitting and can be applied to any ML model. However, this requires additional calculations and consumes more computational time. Tree-based models, on the other hand, calculate FI innately as part of their fitting algorithm, reducing the computational effort needed.^58^

Herein, we utilized decision tree models and two tree-ensemble methods: RF and GB. Tree-based models were selected, as there is no additional computational expense in calculating the FI for these models. Decision trees are branching structures that can be used for prediction based on splitting point (node) rules. In its simplest form, a single decision tree is the predictor. The structure of the tree (node feature selection and threshold value) is tuned by using known data, and either minimizing the GINI impurity (for classification) or the “within-sample” variance (for regression method) at each node.^59,60^ Equations describing how this structure tuning occurs are shown in the methods section.

RF is an ensemble ML technique that combines the prediction of different decision trees, thus minimizing overfitting and data bias.^61^ This combination is also referred to as bagging and considers the predictions of all trees in the forest, either by averaging the final value (for regression) or by majority counting of the selected class (for classification).

GB is another type of tree-ensemble method. Unlike bagging, boosting considers trees in sequence with each new tree building upon the previous one to improve the fit. During tree structure creation and tuning, GB uses the error between the expected and calculated output values instead of the output value itself. The new predicted output value can then be determined from the previous prediction and the new predicted error until the prediction does not change. The mathematical development is explained in the work published by Friedman.^62^

After applying the appropriate ML model to the data set, analysis of the contributions of each input feature can be carried out via FI calculations in tree models and Shapley additive explanations (SHAP) analysis.^63^ FI provides the overall contribution of each feature to the output. However, it does not provide any information on whether the contribution is negative or positive, or on what each feature's contribution is for specific data points. Additional information can be gained by using SHAP values, which provide the marginal contribution of each feature for each data point in the data set, allowing for the identification of features and feature values that lead to higher-performing SAMP designs, as well as clustering of SAMP designs that have similar feature contribution distributions.^63^

In this work, we ran a pilot study, where a ML pipeline was applied to a small set of controlled experimental data with 4 variables. The work utilizes 3 algorithms, decision tree and its ensemble methods, RF, and GB, applied as both regression and classification methods. We explored their potential for the prediction of antimicrobial properties of a library of polyacrylamides. All of the polymers in the experimental data set are polyacrylamides with the same chain-transfer agent (CTA), and *N*-isopropylacrylamide (NIPAM) is used as the apolar monomer of choice to facilitate comparison. The studied SAMPs were designed to vary in the degree of polymerization, architecture, type of cationic moiety, and cationic ratio to enable a broad range of experimental data to be used with the chosen algorithms.

The output variables of the pipeline were (1) the minimum inhibitory concentration (MIC) and (2) hemagglutination, used to predict antimicrobial activity and identify the most significant feature for the optimal selectivity, respectively. As the SAR was also related to the type of bacteria tested, the MIC of each tested strain was used as an output. We trained the algorithms and identified the model with the best performance, which provides reproducible FI results after multiple runs. The aim is to advance our understanding of SARs of antimicrobial polyacrylamide without the need for further experimental work. To the best of our knowledge, such studies have only been conducted recently by Boyer and co-workers,^64,65^ who used a previously published dataset and utilized a decision tree classification model using multiple descriptors to make recommendation for the design of effective SAMPs. Kundi et al. used a library of 147 polymers to understand polymer structure contributions to antimicrobial activity, testing the polymers on one strain of *P. aeruginosa* (PA01).^64^ Although the polymer library used in this work is smaller (23 polymers), the polymers were each tested on 4 bacterial strains (as opposed to 1 Gram-negative by Kundi et al.), including both Gram-positive and Gram-negative bacteria. Additionally, data for hemagglutination were also collected and modelled, instead of MICs only, allowing the models developed here to be applied in multi-objective optimisation to search for polymer designs (within the dataset) that best achieve a compromise in maximising antimicrobial activity across 4 different bacterial strains while minimising agglutination. Finally, SHAP analysis was also performed as in Kundi et al., but datapoint-specific SHAP values were also analysed in addition to dataset-averaged SHAP values. These datapoint-specific SHAP values specifically quantify how much each feature contributed to the best-performing SAMP designs.

## Experimental Section

### Polymer Synthesis and Bioassays

The polymers used in this work (Table 1) were prepared via RAFT polymerization. Their synthesis process and characterization, as well as the MICs against Gram-negative *P. aeruginosa* (PA14 and LESB58) and Gram-positive *S. aureus* (USA300 and Newman) strains and hemocompatibility (C_H_), were all detailed in a previously published study.^44^

**Table 1:** Dataset for All Four MIC Output Variables (μg/mL)

data point #	DP	CatMonPerc	PolyConf^a^	CatMonType^b^	MIC PA14	MIC LESB58	MIC USA300	MIC Newman
1	25	70	2	1	513	128	128	128
2	25	30	2	1	513	512	513	513
3	25	30	2	2	513	513	513	513
4	25	30	2	3	513	513	513	513
5	50	100	1	2	513	513	64	64
6	50	100	1	3	513	513	128	256
7	50	100	2	3	513	513	128	256
8	50	70	2	1	128	64	64	64
9	50	70	2	2	513	513	513	513
10	50	70	2	3	513	513	513	513
11	50	50	2	1	512	512	256	256
12	50	50	4	1	513	513	513	513
13	50	50	2	2	513	513	513	513
14	50	50	2	3	513	513	513	513
15	50	30	3	1	64	256	513	513
16	50	30	2	1	513	512	513	513
17	50	30	4	1	513	513	513	513
18	50	30	3	2	513	513	513	513
19	50	30	2	2	513	513	513	513
20	50	30	3	3	513	513	513	513
21	50	30	2	3	513	513	513	513
22	100	30	2	1	256	128	128	128
23	100	30	4	1	256	128	513	513

a Polymer conformation: 1 = homopolymer; 2 = diblock copolymer; 3 = triblock copolymer; 4 = statistical copolymer.

b Type of cationic monomer: 1 = AEAM; 2 = DMAEAM; 3 = TMAEAM.

### ML Techniques

#### Mapping Input–Output Relationships with RF FI and SHAP Values

ML tools were applied to the data to relate the contribution of each input variable (feature) to the output variable. Separate models were generated for each output (MIC PA14, MIC LESB58, MIC USA300, MIC Newman, and agglutination) based on 4 inputs (type and percentage of cationic monomer, degree of polymerization, and polymer conformation). Three methods were used to create these models: single decision trees, RF, and GB. The complete data sets for model fitting and validation are presented in Tables 1 and 2. It should be noted that categorical inputs (type of cationic monomer and polymer conformation) were assigned a numerical value, and that, while experimental data are available for hemolysis, it was not possible to create models for this output variable because all output values were the same (>512 μg/mL).

**Table 2:** Dataset for Agglutination (μg/mL)

data point #	DP	CatMonPerc	PolyConf^a^	CatMonType^b^	agglut
1′	25	70	2	1	4
2′	25	30	2	1	32
3′	25	30	2	2	256
4′	25	30	2	3	16
5′	50	100	2	3	64
6′	50	70	2	1	4
7′	50	70	2	2	513
8′	50	50	2	1	513
9′	50	50	4	1	128
10′	50	50	2	2	513
11′	50	30	3	1	513
12′	50	30	2	1	32
13′	50	30	4	1	128
14′	50	30	3	2	513
15′	50	30	2	2	256
16′	50	30	3	3	256
17′	50	30	2	3	128

a Polymer conformation: 1 = homopolymer; 2 = diblock copolymer; 3 = triblock copolymer; 4 = statistical copolymer.

b Type of cationic monomer: 1 = AEAM; 2 = DMAEAM; 3 = TMAEAM.

All three methods were tested as classification and regression methods, that is, treating the output variables as categorical or numerical, respectively. Previous studies that modeled polymer property–performance with ML have used classification models.^64,66,67^ However, for the data set in the present work, it was found that classification methods were not reproducible within different runs with distinct training-validation data set splits due to the uneven class distribution in the data set (see Results section and Supporting Information for further discussion). Therefore, regression methods were also tested. For classification methods, continuous output values were assigned either to class 1 (good performance) or to class 0 (undesirable performance), with a continuous output value of 64 μg/mL used as the threshold value. Depending on the output variable, good performance is attributed to values ≤64 μg/mL or >64 μg/mL. For example, for potency, lower concentrations are desired; hence, for all MIC outputs, values ≤64 μg/mL were assigned to class 1. Conversely, it is undesirable to induce hemagglutination at lower concentrations, so values >64 μg/mL were assigned to class 1 in this case.

#### ML Models

For regression decision tree fitting, 20% of the data set was used for testing/validation. For classification tree fitting, the training/testing split was 50% to ensure that both classes were present in the training data set. This was performed in Python with the Scikit-learn package.^58^

The Scikit-learn package for Python was used to create RF ensembles, with out-of-bag cross-validation and hyperparameter optimization.^58^ 20% of the data points were randomly selected with replacement and used to validate each tree structure in the forest for the regression method. As the data set is small, cross-validation was chosen instead of splitting between training-test data sets for regression. The split was 50% for classification. Fitting was repeated 5 times to ensure reproducibility.

Similar to the other two methods, GB was implemented in Python with Scikit-learn, using 20% of the data set for cross-validation with the regression method, and a 50% train-test split for classification.

#### FI and SHAP Values

SHAP values calculations were implemented in Python with the SHAP package.^68^ SHAP was used to explain how each feature contributed to the predicted output values of the best previously generated models. More information about how FI is calculated for tree models is given below.

#### Tree Models and FI

Figure 1 shows a split across a node t in a decision tree *T*, for continuous output data. When fitting the tree structure, the goal is to minimize the impurity across the node. This is accomplished by selecting which feature the node is splitting on (*X_n_*) and what threshold value is used for the split condition (*s*).

**Figure 1:**
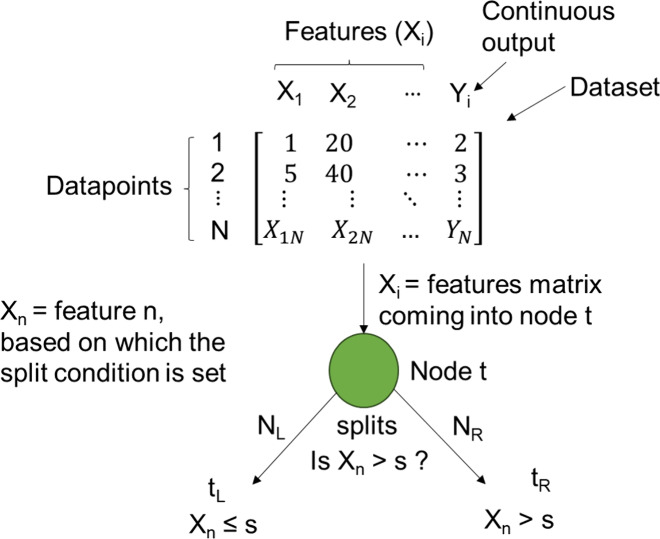
Sample node *t* within a tree *T*. Coming into node *t* is matrix *X_i_*, containing information about the features (input variables). If node *t* is the first node in the tree, then this matrix contains all *N* data points in the data set. Otherwise, matrix *X_i_* contains a subset that comes from a previous node split. *X_n_* is the feature over which the data set splits, and *s* is the threshold value for this split. A subset of matrix *X_i_* (with *N*_L_ data points) splits to the left at node *t* split *t*_L_, for the data points in which feature *X_n_* has a value equal to or smaller than the threshold *s*. Another subset of *X_i_* (with *N*_R_ data points) splits at *t*_R_, for values of *X_n_* greater than the threshold. Within a tree *T*, the selection of *X_n_* and *s* for each node split is achieved by minimizing the impurity of the node with respect to the output variable, *Y_i_*.

The concept of impurity comes from classification trees, where impurity represents the degree to which data points have been incorrectly classified at any node. For regression trees, impurity is treated as the variance, or “within-sample” variance,^69^ as shown in the equations below.

The impurity (Δ̂) coming into node t is given by

$$\mathrm{Impurity}\;\mathrm{into}\;\mathrm{node}\;t,\;\hat{\Delta }(t)=\frac{1}{N}\sum_{X_{i}\in t}(Y_{i}-\overset{®}{Y_{t}}{)}^{2}$$

where *N* is the number of samples (data points) in node *t*; *Y_i_* is each output value present in the data set coming into node *t*; and $\overset{®}{Y_{t}}$ is the sample mean for the output variable for data points coming into node *t*.

The impurity coming out of node *t* is

$$\mathrm{Left}:\;\hat{\Delta }(t_{L})=\frac{1}{N_{L}}\sum_{X_{i}\in t_{L}}(Y_{i}-\overset{®}{Y_{L}}{)}^{2}$$

$$\mathrm{Right}:\;\hat{\Delta }(t_{R})=\frac{1}{N_{R}}\sum_{X_{i}\in t_{R}}(Y_{i}-\overset{®}{Y_{R}}{)}^{2}$$

The variables are similar to the ones coming into the node, except now *N*_L_ and *N*_R_ are the number of data points present in the left and right splits of the node, respectively; *Y_i_* is each output value in the part of the incoming data set that was split into either the left or the right split; and $\overset{®}{Y_{t}}$ and $\overset{®}{Y_{t}}$ are the mean output values for data points in the left and right splits, respectively.

The decrease in impurity across node *t* is given by the difference in impurity coming in and out, with the impurity coming out weighted by the fraction of samples that go into each split.

$$\hat{\Delta }(s,t)=\hat{\Delta }(t)-[\hat{p}(t_{L})\hat{\Delta }(t_{L})+\hat{p}(t_{R})\hat{\Delta }(t_{R})]$$

where

$$\begin{array}{c} \hat{p}(t_{L})=\frac{N_{L}}{N}\\ \hat{p}(t_{R})=\frac{N_{R}}{N} \end{array}\}\mathrm{Fraction}\;\mathrm{of}\;\mathrm{samples}\;\mathrm{that}\;\mathrm{go}\;\mathrm{into}\;\mathrm{each}\;\mathrm{split}$$

To minimize impurity, the algorithm maximizes Δ̂(*s*, *t*), which is the *decrease* in impurity.

For classification trees, “within-sample” variance is replaced by the GINI impurity (given below) in the equations above.

$$\mathrm{GINI}\;\mathrm{impurity}={\left(1-\sum_{i}^{K}P_{i}^{2}\right)}_{j}$$

where *K* is the total number of classes present (usually two for a binary classification problem), *i* is each class, and *P_i_* is the probability of observing class *i* in split *j*.

FI for each node *t* (*I*_node,*t*_) can be calculated directly from each split's impurity. This is shown below for a classification tree.^70^

$$I_{\mathrm{node},t}=w_{t}{\left(1-\sum_{i}^{K}P_{i}^{2}\right)}_{t}-\sum_{j}^{N}w_{j}{\left(1-\sum_{i}^{K}P_{i}^{2}\right)}_{j}$$

where *t* is the node; *w_t_* is the weight of the sub-dataset coming into node *t* (that is, the fraction of the total data points in the data set that belong to this sub-dataset); subscript *t* represents the sub-dataset coming into the node; *w_j_* is the weight of the splits coming out of node *t* (for the usual case where each node is a bifurcation, *N* = 2, and *j* = 1 or 2, left or right); and subscript *j* represents each split out of node *t*.

The importance of each feature *n* can then be calculated by summing the importance of all nodes that split based on feature *n* and then dividing by the sum of the importance of all nodes.

$$I_{\mathrm{feature},n}=\frac{\sum_{t\;\mathrm{that}\;\mathrm{splits}\;\mathrm{on}\;\mathrm{feature}\;n\;\mathrm{only}}I_{\mathrm{node},t}}{\sum_{\mathrm{all}\;t}I_{\mathrm{node},t}}$$

FI is then normalized between 0 and 1.

$$\mathrm{norm}I_{\mathrm{feature},n}=\frac{I_{\mathrm{feature},n}}{\sum_{n}I_{\mathrm{feature},n}}$$

For bagging ensemble methods, such as RFs, the combined FI over all trees is given by^58^

$${\mathrm{RFI}}_{\mathrm{feature},n}=\frac{\sum_{T}(\mathrm{norm}I_{\mathrm{feature},n}{)}_{T}}{N_{T}}$$

where *T* is each tree in the forest and *N_T_* is the total number of trees.

#### Model Fit and Consistency Testing

To select the best-performing model(s), all three methods (decision tree, RF, and GB) as both regression and classification, were fit in 5 different runs (i.e., with different random selections for training and testing data sets) while optimizing some of each method’s hyperparameters. Hyperparameters that affect the size of the trees and forests were chosen for optimization (such as the maximum tree depth and number of estimators). Hyperparameters related to the mathematical formulation of the algorithm were left at their default values, and those related to the selections made in regard to testing and validation splits, cross-validation, and bagging were set to their specific values as mentioned previously in this section. It should be noted that tuning different hyperparameters can lead to similar model results, and in this case, the hyperparameter tuning that gives the smaller training error was chosen.^58^

The fits of each method (expressed as the *R* ^2^ value for regression and precision, recall, and F1 score metrics for classification) were considered when selecting the method that would be the best for representing the polymer property–performance space. However, as it was noticed that some methods exhibited different FI distributions among different runs, or different tree structures for single decision tree and the final GB tree, the ability of each method to remain consistent across different runs was also factored into the model selection decision.

## Results

### Polymer Library

A total of 23 polymers (Table 1) were utilized with NIPAM as the apolar monomer of choice for all copolymers and cationic monomers based on (1) the primary amine ethyl acrylamide (AEAM), (2) the tertiary amine dimethyl ethyl acrylamide (DMAEAM), and (3) the quaternary amine trimethyl ethyl acrylamide (TMAEAM). The cationic monomer ratios were 30, 50, 70, and 100%. The polymer characterization results including ^1^H NMR and GPC, the MICs against *P. aeruginosa* and *S. aureus* strains, and hemagglutination (C_H_) concentrations were all determined in a previously published work.^44^

Briefly, the results highlight the significance of the cationic charge in determining the activity and selectivity of polymers. Using the primary amine in a high ratio (70%) in the diblock copolymer enhanced the antimicrobial profile but reduced the selectivity. The polymer architecture also influenced the potency; the AEAM-triblock (DP50-Ta30) induced a similar potency toward Gram-negative bacteria strains at a lower cationic ratio (30%) compared to the diblock (DP50-Da70). The methylation of the cationic units of the block copolymers diminished their potency. Only homopolymers of DMAEAM and TMAEAM were active against Gram-positive bacteria.

### ML Model Selection

Model selection considered both the fit and reproducibility of the FI. Simpler, linear models were tested (Figure S1), but failed to appropriately capture the highly non-linear, complex nature of the dataset (fitting metrics and parity plot are shown in the Supporting Information). This confirms the choice of training the more complex ML models discussed below.

### Fit and Predictability of the ML Methods

For each method used to create models (decision tree, RF, and GB), the fit was evaluated as the *R* ^2^ value for regression methods (Table 3) and the precision, recall, and F1 scores for classification methods (Table 4).

**Table 3:** Fit Metrics of the Regression Models

method	output variable	*R* ^2^ ^a^	MAE	RSME	MedAE	Output Range
decision tree	MIC PA14	1.00^+^	0	0	0	64-513
	MIC LESB58	1.00^+^	0	0	0	64-513
	MIC USA300	1.00^+^	0	0	0	64-513
	MIC Newman	1.00^+^	0	0	0	64-513
	agglutination	1.00^+^	0	0	0	4-513
random forest	MIC PA14	0.82	33	56	13	64-513
	MIC LESB58	0.90	31	42	18	64-513
	MIC USA300	0.88	42	62	30	64-513
	MIC Newman	0.89	36	55	20	64-513
	agglutination	0.83	52	72	46	4-513
gradient boosting	MIC PA14	0.98	12	20	6	64-513
	MIC LESB58	1.00	5	6	3	64-513
	MIC USA300	0.99	12	13	8	64-513
	MIC Newman	0.99	11	12	8	64-513
	agglutination	1.00	1	1	1	4-513

a *R* ^2^: for decision trees, as a single tree is fit, 20% of the sample data points were randomly selected for validation, so two *R* ^2^ values were obtained, one for training and one for validation. For RF and GB, it was possible to perform cross-validation (out-of-bag sampling with replacement) for each tree in the ensemble, so a single *R* ^2^ is reported for the ensemble. ^**+**^: *R* ^2^ values of exactly 1 were obtained for both training and validation data sets. MAE: mean absolute error. RSME: root-mean squared error. MedAE: median absolute error.

**Table 4:** Fit Metrics of the Classification Models

method	output variable	precision^a^		recall^a^		F1 score^a^	
decision tree	MIC PA14	1	1	1	1	1	1
	MIC LESB58	1	0.5	1	0.46	1	0.48
	MIC USA300	1	0.45	1	0.41	1	0.43
	MIC Newman	1	0.44	1	0.36	1	0.4
	agglutination	1	0.61	1	0.58	1	0.58
random forest	MIC PA14	1	1	1	1	1	1
	MIC LESB58	1	1	1	1	1	1
	MIC USA300	1	0.46	1	0.5	1	0.48
	MIC Newman	1	0.46	1	0.5	1	0.48
	agglutination	1	0.75	1	0.86	1	0.75
gradient boosting	MIC PA14	1	1	1	1	1	1
	MIC LESB58	1	0.5	1	0.38	1	0.43
	MIC USA300	1	0.45	1	0.45	1	0.45
	MIC Newman	1	0.5	1	0.95	1	0.81
	agglutination	1	0.67	1	0.5	1	0.44

a Precision, recall, and F1 score are given as macro-average values (simple mean between the values for each class); the first value is for the training data set and the second for the validation data set. The precision, recall, and F1 score for each class are shown in the Supporting Information.

Classification models obtained good fits on the training data set (achieving precision and recall of 1 for all methods and output variables) but failed to perform well on the testing data set. Very few data points of MIC output variables (1 or 2) were available for class 1, which limited the fitting quality of the testing data set (see Supporting Information). Furthermore, values of 1 for precision, recall, and F1 score of the testing data set (Table 4) may give an incorrect sense of good fit 1 (see Supporting Information). However, there was an almost even split of classes for hemagglutination (H_c_) output, although the fit of the testing data set was marginally better than that observed for MIC output variables for decision trees and RF models (i.e., a higher F1 score).

For these reasons, it was decided to continue with the modeling approach using regression models.

Regression models achieved good fits (Table 3). For decision trees, the fits to both training and testing (validation) data sets were exactly 1, while for RF and GB, a single *R* ^2^ value is used to represent the fit, as cross-validation was performed during fitting and indicates good fits as well. Therefore, we plotted the actual value versus the predicted value for regression RF and GB models (Figure 2), and the regression GB model has better predictability (Figure 2A). Residual plots for both models are shown in the Supporting Information (Figure S1). Residual (error) metrics (Table 3) also indicate a perfect fit for the decision tree models (however, tree reproducibility was poor, as discussed further in the text), and good fits for random forest and, especially, gradient boosting models.

**Figure 2:**
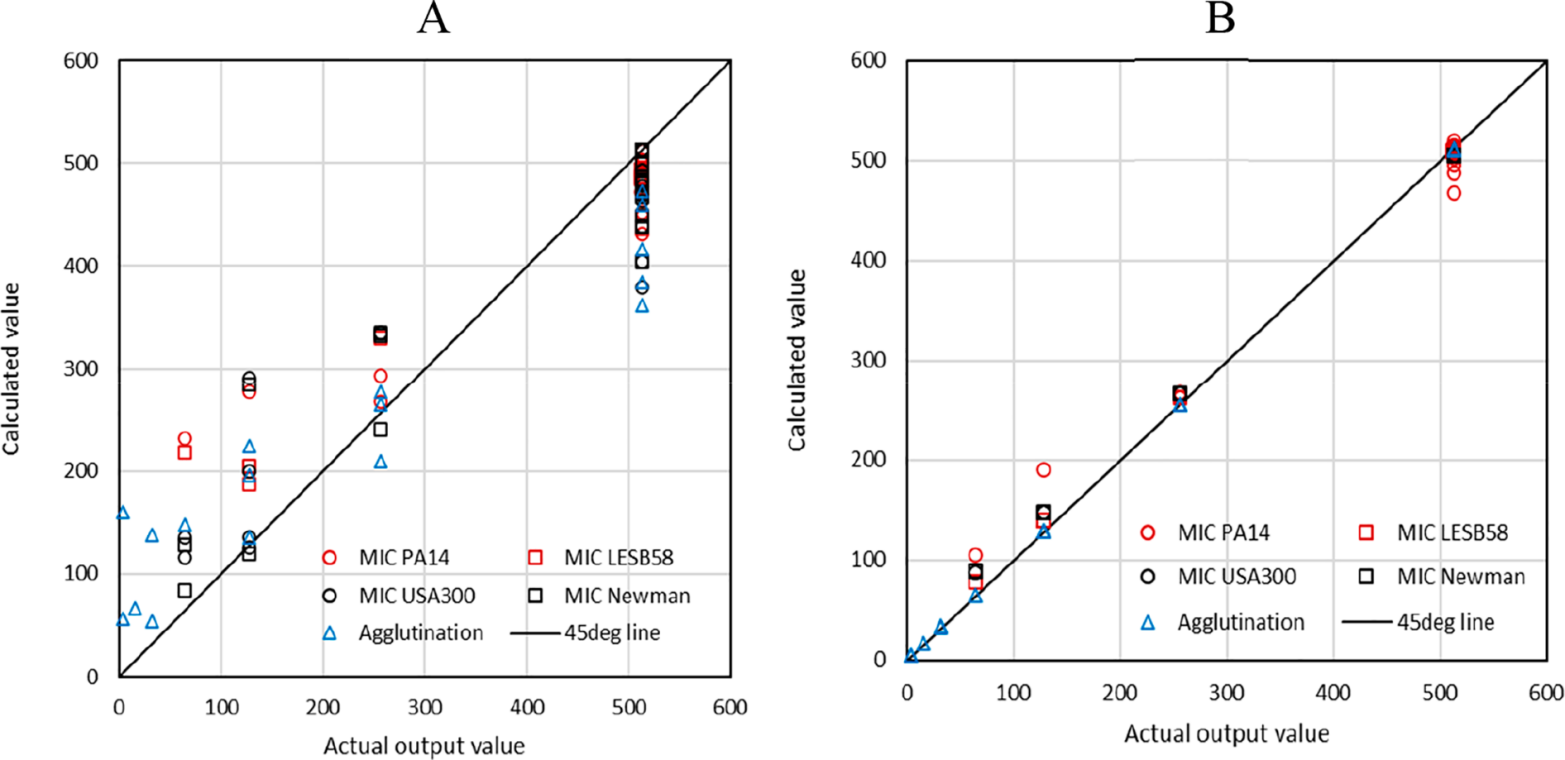
Parity plots for A) RF regression models (left) and B) GB regression models (right). The model-predicted output values are plotted against the actual values for all five models: MIC PA14 (red open circles), MIC LESB58 (red open squares), MIC USA300 (black open circles), MIC Newman (black open squares) and Agglutination (blue open triangles). A 45-degree line is provided as a reference.

In addition to considering the model fit, reproducibility of FI and consistency of the generated tree structure were included in the analysis for the selection of the best-performing model(s).

### Reproducibility and Consistency of the ML Models

The reproducibility of FI between different models was tested by performing 5 runs in which distinct data points were selected for validation/cross-validation and with separate hyperparameter optimizations (with 100 random trials). The average FI for each feature and each output variable is shown in Table 5 for regression models (the data for classification models is shown in the Supporting Information). The optimized hyperparameter values, fitting metrics, and individual FI values for each run can be seen in the Supporting Information.

**Table 5:** Average FI, Standard Deviation, and Error for Regression Models^a^

		decision tree			random forest			gradient boosting		
output variable	feature	avg	Stdev	Stder (%)	avg	Stdev	Stder (%)	avg	Stdev	Stder (%)
MIC PA14	*DP*	0.21	0.02	4	0.38	0.07	8	0.16	0.03	7
	*CatMonPerc*	0.16	0.16	46	0.16	0.04	12	0.26	0.01	1
	*PolyConf*	0.41	0.26	28	0.22	0.05	9	0.33	0.02	2
	*CatMonType*	0.22	0.10	21	0.24	0.04	8	0.25	0.00	1
MIC LESB58	*DP*	0.29	0.17	27	0.33	0.05	6	0.36	0.00	0
	*CatMonPerc*	0.25	0.10	19	0.25	0.03	5	0.37	0.00	0
	*PolyConf*	0.07	0.08	52	0.10	0.04	16	0.10	0.00	0
	*CatMonType*	0.39	0.25	29	0.32	0.02	3	0.17	0.00	0
MIC USA300	*DP*	0.09	0.06	29	0.12	0.02	6	0.07	0.00	0
	*CatMonPerc*	0.63	0.10	7	0.60	0.01	1	0.52	0.00	0
	*PolyConf*	0.17	0.11	30	0.13	0.01	5	0.14	0.00	0
	*CatMonType*	0.11	0.07	30	0.15	0.02	6	0.26	0.00	0
MIC Newman	*DP*	0.08	0.09	49	0.12	0.02	9	0.09	0.00	0
	*CatMonPerc*	0.50	0.10	9	0.55	0.03	2	0.57	0.00	0
	*PolyConf*	0.16	0.05	13	0.14	0.01	4	0.17	0.00	0
	CatMonType	0.27	0.09	16	0.19	0.02	5	0.18	0.00	0
Hc	*DP*	0.07	0.10	69	0.14	0.03	8	0.18	0.00	0
	*CatMonPerc*	0.40	0.09	10	0.26	0.04	6	0.22	0.00	0
	*PolyConf*	0.23	0.12	24	0.26	0.03	5	0.20	0.00	0
	*CatMonType*	0.31	0.11	16	0.35	0.02	3	0.40	0.00	0

a Avg = average value; Stdev = standard deviation (5 runs); Stder (%) = percent standard error.

For regression models, the decision tree had the highest standard error among all runs, averaging 20–34% for the different output values. RF and GB had average standard errors ranging between 4 and 9% and 0–3%, respectively. Classification models (Table S4 in the Supporting Information) exhibited larger standard errors: 28–51% for decision tree, 17–37% for RF, and 23–64% for GB.

Regression RF and GB models were the most reproducible, and this can be more easily visualized by plotting the FI of different runs in spider plots (Figure 3). The plots for all other regression and classification models are given in the Supporting Information (Figures S2–S6).

**Figure 3:**
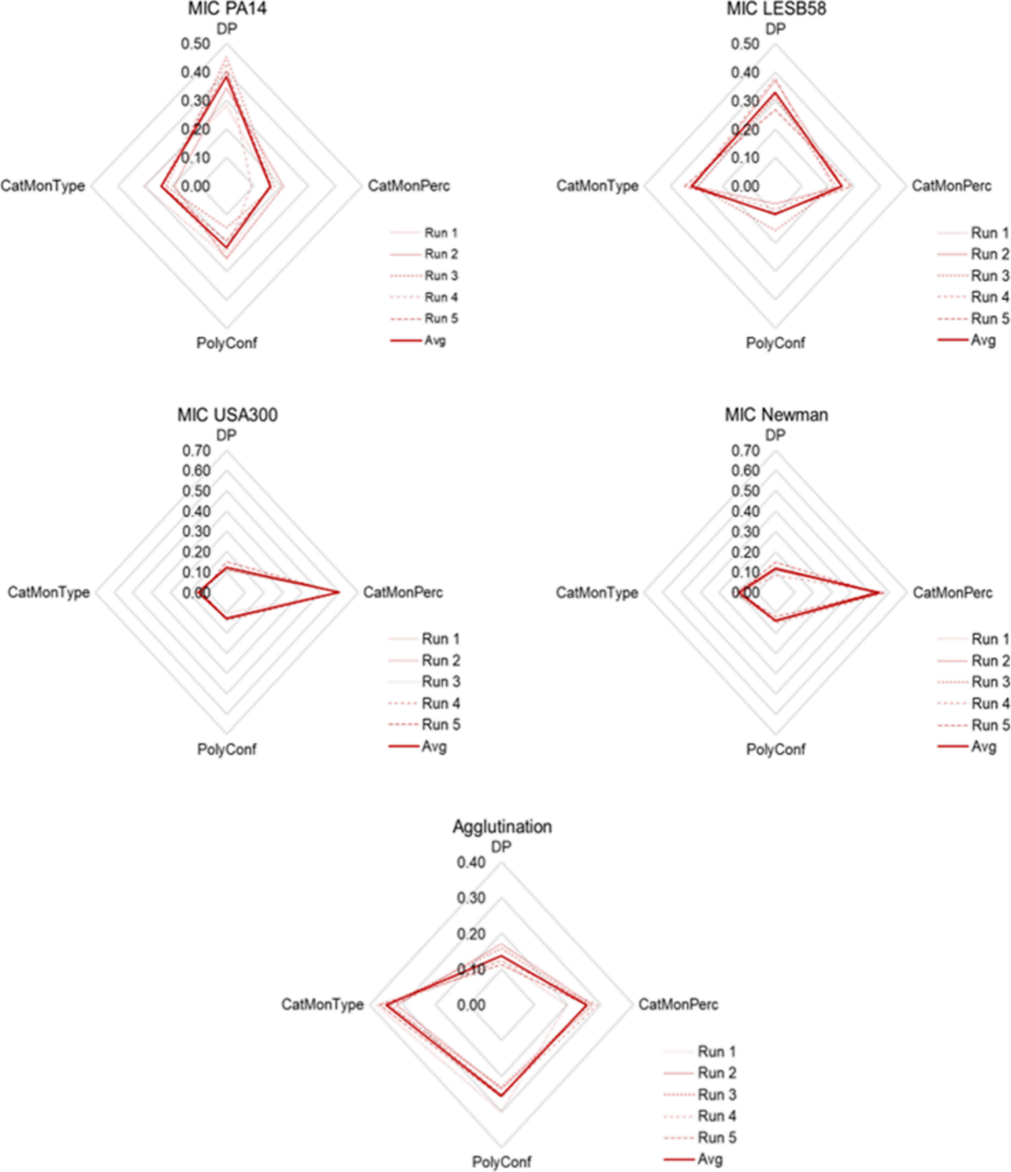
Reproducibility test of FI for regression RF models. For each output variable (MIC PA14, MIC LESB58, MIC USA300, MIC Newman, and agglutination), five different runs were performed, and the importance of each feature (degree of polymerization [DP], type of cationic monomer [CatMonType], percentage of cationic monomer [CatMonPerc], and polymer confirmation [PolyConf]) was calculated for each run. The average across all of the runs is also shown. In tree-based models, FI is computed during model fitting and derived from node impurity calculations.

The consistency of the tree structure between different runs was also analyzed. For RF models, tree structures are individually less impactful given that the average of all trees’ predictions is taken into account during bagging. This analysis is discussed in detail in the Supporting Information. Briefly, the decision tree models mostly fit distinct tree structures across different runs, and this resulted in high variability of the average FI. For GB regression models, while the initial trees in the sequence presented slight structural changes between runs, the final trees were either identical or had node splits at deeper levels (which have a smaller impact on FI).

In general, the classification models had low fitting scores at the testing stage of the data set and with inability to reproduce the average FI distributions after multiple runs. On the other hand, RF and GB regression models fit the data well and were reproducible across distinct runs. This indicates that these two models best capture the input–output relationships of the data and were therefore applied to further ML data analysis.

### FI and Average SHAP Values

Based on both model fits, FI reproducibility, and consistency of tree structures, as discussed previously, the regression RF and GB models were selected for representing the data set and further SHAP analysis.

### FI for RF and GB Regression Models

From the average FI values (Table 5 and Figure 3), both RF and GB models predicted similar FI distributions for MIC USA300 and MIC Newman, with the percentage of the cationic monomer being the greatest contributing input (feature), with 50–60% contribution, followed by the type of cationic monomer and polymer conformation, and finally the degree of polymerization, with less than 20% contribution.

The MIC for *P. aeruginosa* strains exhibited variable feature contributions for both the models and the strains. Based on the RF regression model, the order of feature contributions for MIC PA14 was DP > type of cationic monomer > polymer conformation and cationic monomer ratio. However, for MIC LESB58, the order was similar but the DP and type of cationic monomer were the features contributing the most, close to around 30%, followed by the cationic ratio and polymer conformation with about 10% contribution.

With the GB regression model, for MIC PA14, more importance was placed on polymer conformation (33% versus 22%) and percentage of cationic monomer (26% versus 16%) rather than on DP (16% versus 38%), relative to the RF model. For MIC LESB58, the GB model placed more importance on the percentage of cationic monomer (37% versus 25%), but the importance was relatively the same for DP and polymer conformation. The GB model also placed less importance on the type of cationic monomer (17% vs 32%) than RF.

For agglutination, RF and GB placed similar levels of importance on the same features. The type of cationic monomer was the most important feature (35–40%), followed by polymer conformation (22–26%) and the cationic monomer ratio (26–20%).

### Comparing FI and Average SHAP Values for RF and GB Regression Models

SHAP values explain how each feature impacts the expected output values in a data set. While FI only provides the magnitude of the contribution for the whole data set, SHAP provides the magnitude and the type of contribution (positive or negative) for each data point in the data set. The advantage of FI is that it is calculated as part of the tree fitting procedure (refer to the equations presented earlier in the Experimental Section), and no additional computational work is needed to obtain those values. SHAP values were averaged over the data set and compared to the FI (Figure 4). The values in the graphs are given in the Supporting Information (Tables S17 and S18).

**Figure 4:**
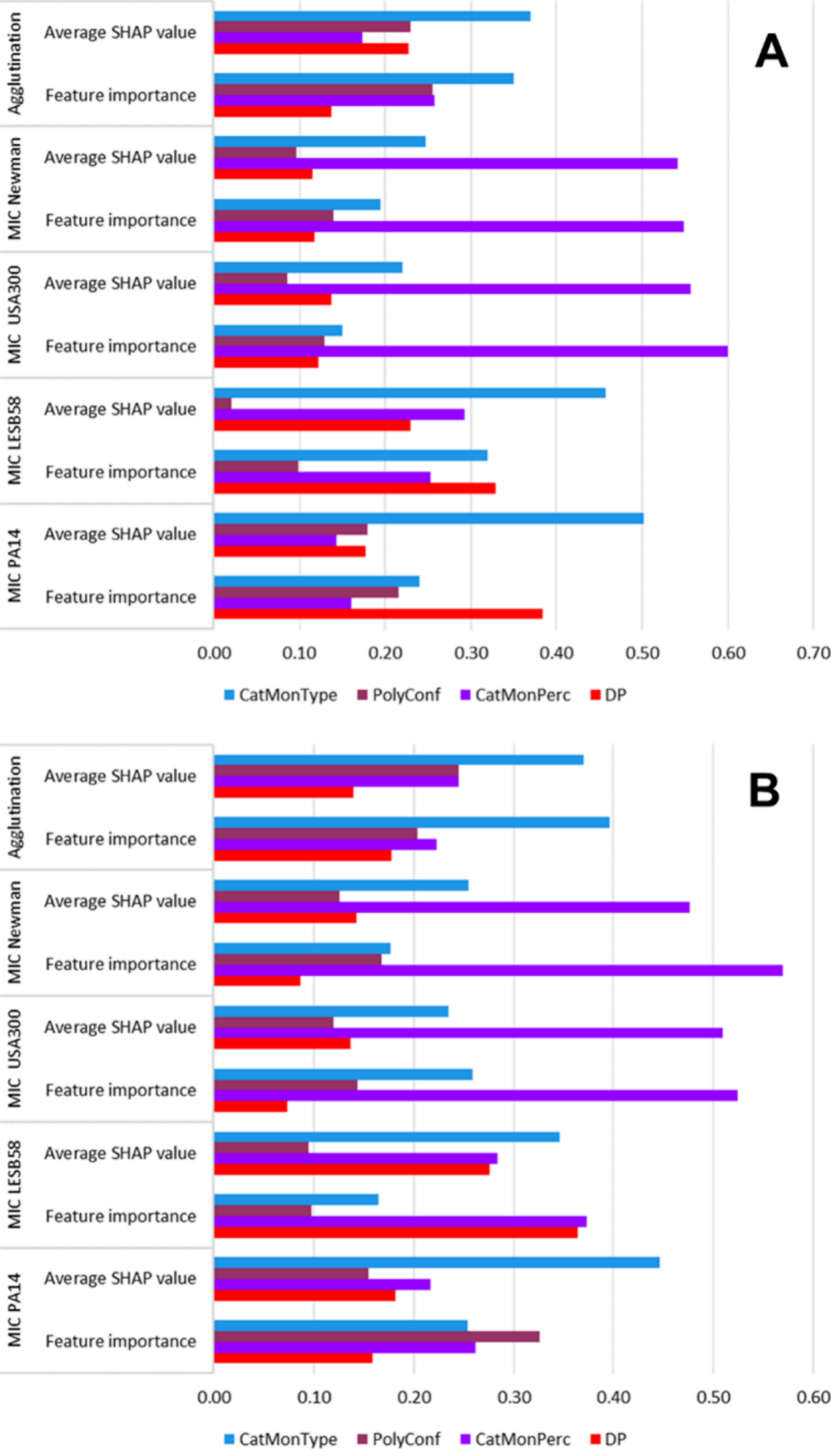
Comparison of FI and average SHAP values for all output variables (MIC PA14, MIC LESB58, MIC USA300, MIC Newman, and agglutination). The importance and SHAP values of each model feature (input variable)—degree of polymerization (DP), type of cationic monomer (CatMonType), percentage of cationic monomer (CatMonPerc), and polymer confirmation (PolyConf)—are shown on a scale of 0–1, and these values add up to 1. This comparison is illustrated for the RF regression (A) and GB regression (B) models.

In the RF model, the average SHAP value places less importance on DP and more importance on the type of cationic monomer than FI for both MIC PA14 and MIC LESB58. The importance of the polymer conformation and percent of cationic monomer remained similar for MIC PA14, while for MIC LESB58, the importance of the polymer conformation was minimal with the average SHAP value. For the other output variables (MIC USA300, MIC Newman, and agglutination), FI and average SHAP values were equivalent and there was not much variation in their distributions.

With the regression GB model, SHAP values were also similar to FI distributions for MIC USA300, MIC Newman, and agglutination. For MIC PA14, the average SHAP value allocated greater importance to the type of cationic monomer and reduced importance to the polymer conformation relative to the average FI. For MIC LESB58, the average SHAP also placed more importance on the type of cationic monomer but less importance to the degree of polymerization and percentage of cationic monomer.

Comparing the average SHAP values from the RF and GB models, it should be noted that their distributions are more similar than those of FI from the same RF and GB models. The average SHAP values from different models give more consistent results for the overall data set than FI.

### Data-Point-Specific SHAP Values

SHAP values for each data point represent the marginal contribution of each feature relative to the average output value for the whole data set. In other words, the SHAP value indicates how much each feature contributes to increase or decrease a data point’s output compared to the average output value. We visualized the SHAP values via waterfall curves and beeswarm plots.

In the beeswarm plots (Figure 5), each dot on the plot represents one data point. The *x*-axis represents the SHAP value. A value of zero means that the data point does not contribute to changing the output value. A negative value means that the feature decreases the value of the output, while a positive value means that it increases it, all in relation to the average value of the variable. The average values between the two models (Table 6) are slightly different, as each model provides a different estimate for each data point. The colors of the dots are related to the feature value with the color bar on the right. For features that are inherently categorical but treated as continuous during modeling, the high–low grading can be related to the original feature class by referring to Tables 1 and 2. For example, low CatMonType corresponds to cationic monomer 1 (AEAM), and medium-high PolyConf corresponds to polymer conformation 3 (triblock copolymer).

**Table 6:** Dataset-Averaged Output Values, *E*[*f*(*x*)], in μg/mL for the RF and GB Regression Models

output variable	RF regression	GB regression
MIC PA14	451.8	454.3
MIC LESB58	434.0	432.0
MIC USA300	393.3	395.8
MIC Newman	411.5	407.0
agglutination	217.3	227.6

**Figure 5:**
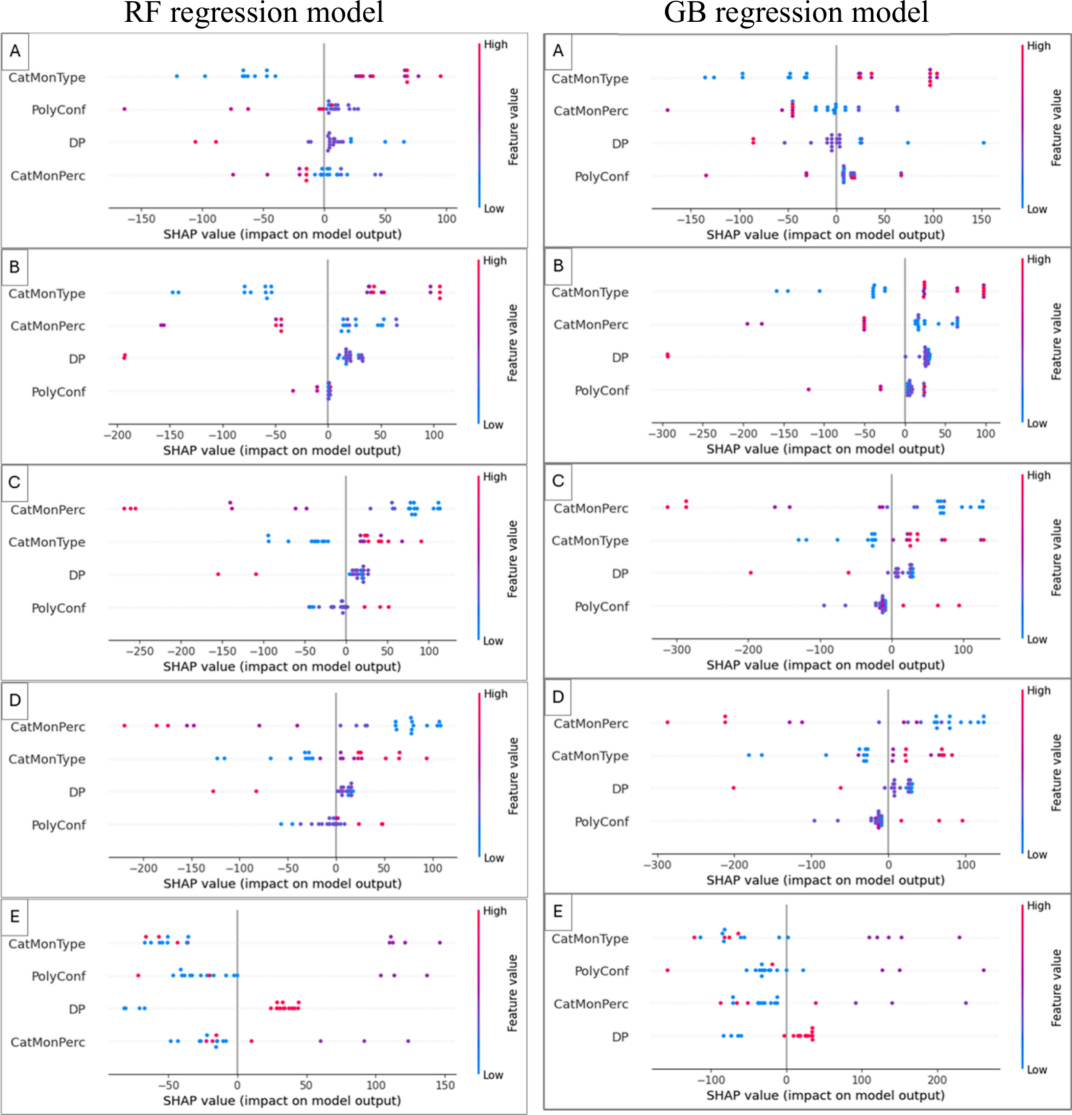
Beeswarm plots for the RF (left) and GB (right) regression models. The color bar gives the magnitude of continuous features and indicates the class for categorical features. (A) MIC PA14; (B) MIC LESB58; (C) MIC USA300; (D) MIC Newman; (E) Hemagglutination.

Therefore, the beeswarm plots can be used to identify feature values that contribute to moving the output closer to the desired value. For all four MIC outputs, a small output value is desirable, as this means greater potency. In contrast, higher output values are desirable for hemagglutination (C_H_).

Comparing the beeswarm plots for RF and GB models (Figure 5), the SHAP value spread is similar for all outputs. While there are some differences in the magnitude of the SHAP values, the color distribution of the dots remains the same for each output variable. For example, focusing on each of the four features for MIC PA14 and specifically on the negative side of the *x*-axis (Figure 5A), it can be seen that the feature values leading to negative SHAP are low for the type of cationic monomer, high for the DP, medium-high for the polymer conformation, and medium-high to high for the cationic monomer ratio (although some low CatMonPerc values are also slightly negative). According to this, the DP 100 triblock copolymer with over 50% AEAM will result in the lowest MIC PA14, for example. A similar analysis was performed for the other outputs, and the results are summarized in Table 7.

**Table 7:** Feature Values that Contribute to Moving the Output Closer to Its Desired Value

	DP		CatMonPerc		PolyConf		CatMonType	
MIC PA14	high	100	medium-high to high^b^	70–100%	3	triblock	1	AEAM
MIC LESB58	high	100	medium-high to high	70–100%	3	triblock	1	AEAM
MIC USA300	high	100	medium-high to high	70–100	2^c^	diblock	1	AEAM
MIC Newman	high	100	medium-high to high	70–100%	2^c^	diblock	1	AEAM
C_H_	medium^a^	50	medium	50%	2	diblock	2	DMAEAM

a Comparing values in Tables S1 and S2, the color bar for agglutination (C_H_) is on a different scale compared to the MIC, as fewer data points were available. Therefore, high DP in the agglutination plot corresponds to a similar value as medium DP in MIC plots.

b Some low CatMonPerc points are also favorable, although not as much as medium-high to high ones.

c Conformations 1 and 2 are favorable based on RF models, while conformation 2 is the most favorable based on GB models (with conformations 1 and 3 also favorable, but not as much as conformation 2).

It is worth mentioning that the most visible difference in SHAP value spread occurs for the polymer conformation feature in MIC USA300 and MIC Newman models (Figure 5C,D). While for RF models, homopolymer and diblock (polymer conformations 1 and 2 in blue and light-purple dots) are more favorable (i.e., more negative), for GB models, diblock (polymer conformation 2) has the most negative SHAP value and thus is the most favorable. Conformations 1 (as in RF) and 3 (homopolymer and triblock) also have negative SHAP values and are favorable.

Caution should be taken when interpreting beeswarm plots, as the features are considered separately, so any potential correlations between features could have been overlooked. These potential correlations may be observed when looking into the combined effect of the SHAP values of all features on specific data points. For this purpose, waterfall plots are used.

Waterfall plots (Figures 6–8) are data-point-specific and show how each feature contributes (both in magnitude and direction) to taking the output value from the sample-averaged value (*E*[*f*(*x*)]) to the predicted output value (*f*(*x*)) for the selected data point. Here, we will discuss the waterfall plots for the best-performing data points in each output variable (MIC ≤ 64 μg/mL and agglutination concentration > 256 μg/mL).

**Figure 6:**
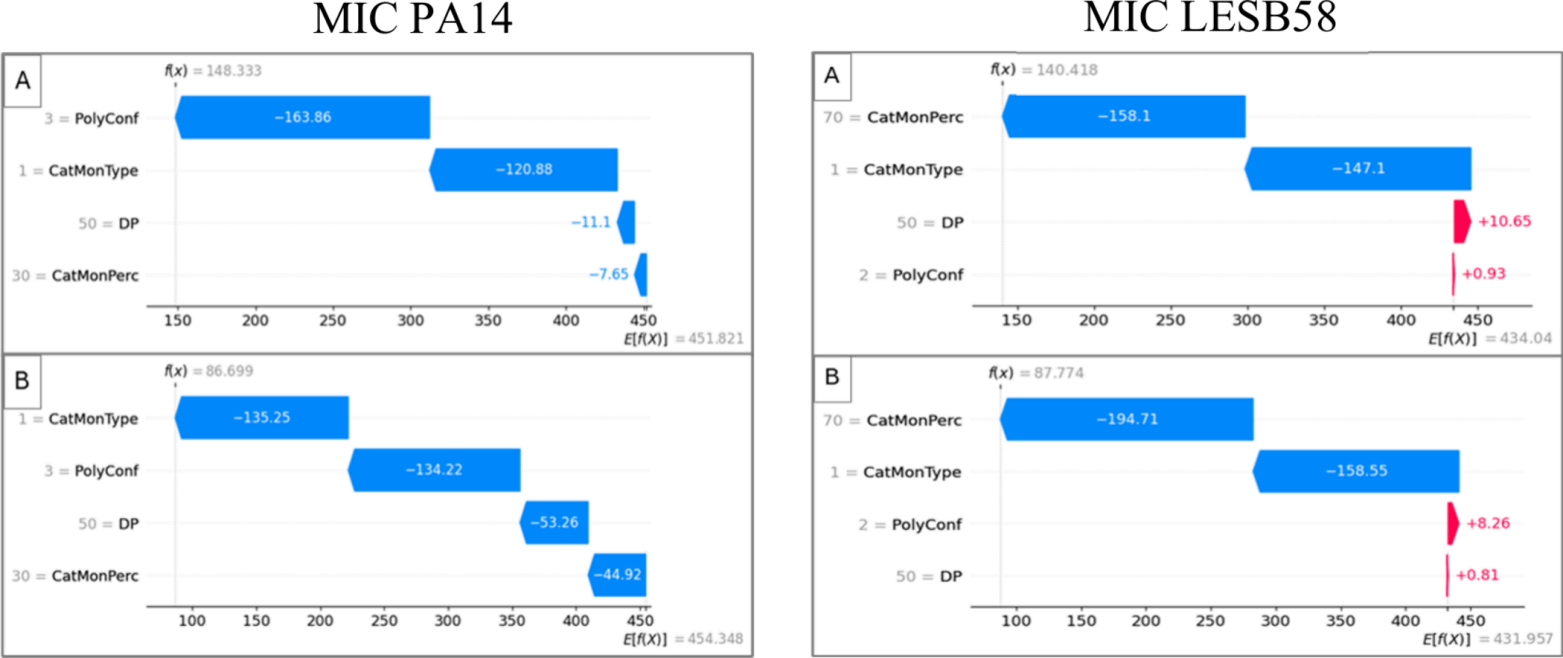
Waterfall plots for best-performing MIC PA14 (data point 15) and MIC LESB58 (data point 8) SAMP designs for each model: A (RF model) and B (GB model). Waterfall plots depict data-point-specific SHAP values. Reading the plots starts at the data set-averaged output value (*E*[*f*(*x*)], as shown in Table 6). Then, each feature’s SHAP value (identified by red arrows if positive and blue arrows if negative) adds to or subtracts from the data-set-averaged output value, giving as the final answer the data point-specific output value (*f*(*x*)) for a given set of feature values.

The waterfall plots for the best-performing MIC PA14 data point obtained from both RF and GB models are shown in Figure 6. For both models, the data-point-specific SHAP values suggest that polymer conformation 3 (triblock) and the type of cationic monomer 1 (AEAM) are the largest contributors to reducing the MIC value. The GB model places more contribution on the other two features, leading to a predicted value (86.7 μg/mL) closer to the actual MIC (64 μg/mL).

Interestingly, while based on the beeswarm plots (Figure 5), high DP was predicted to be beneficial, the best-performing data point in the waterfall plot was a medium DP value (DP50). This illustrates how combined with polymer conformation 3 (triblock) and cationic monomer 1 (AEAM), a lower DP can be favorable. However, it should be noted that a data point with a higher DP and these same conformation and cationic monomer values were not included. This highlights the importance of investigating the relationships between features to further inform, confirm, or challenge insights gained from a beeswarm plot.

Similar to MIC PA14, MIC LESB58 had only one best-performing data point, data point 8 (Figure 6). The type of cationic monomer was also AEAM (1), but different from MIC PA14, LESB58 had the most beneficial contribution from the percent of cationic monomer at a medium-high value (70%).

Two data points performed well for MIC USA300 (data points 5 and 8). For both data points and in both models (Figure 7), the cationic monomer percent was the most favorable feature, at a medium to high value (70–100%). Homopolymers and diblock copolymers were both favorable (classes 1 and 2). The AEAM monomer was the second-best contributor. Both models explained the data point performance in similar ways, with only a small difference in the DP SHAP value for data point 8 (a slightly positive value for RF, but slightly negative for GB). MIC Newman followed similar trends (Figure 7), except for data points 5 and 8. For the GB model data point 5, the type of cationic monomer was the second most contributing feature instead of the polymer conformation. For data point 8, the type of cationic monomer contributed more than the percent of cationic monomer. However, both models and data points considered the percentage of cationic monomer at medium-high amounts (70%) and cationic monomer AEAM as the two most contributing features, similar to MIC USA300.

**Figure 7:**
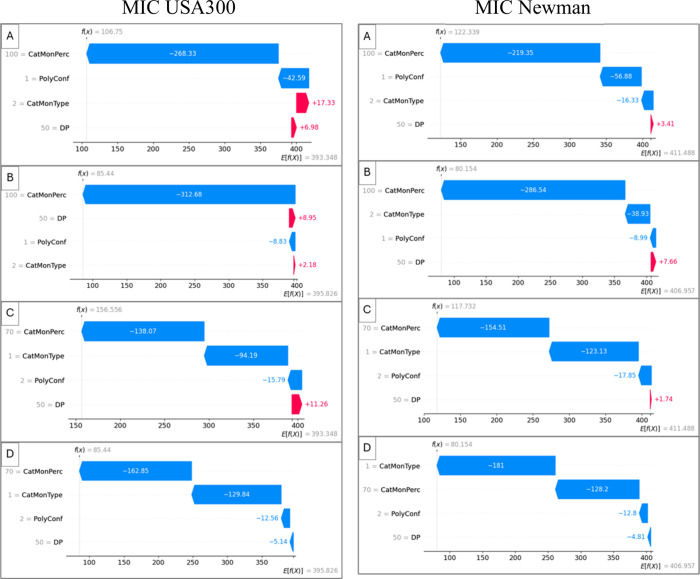
Waterfall plot for best-performing MIC USA300 (left) and MIC Newman (right) SAMP designs (data points 5 and 8). (A) RF model data-point-specific SHAP values for data point 5; (B) GB model data-point-specific SHAP values for data point 5; (C) RF model data-point-specific SHAP values for data point 8; (D) GB model data-point-specific SHAP values for data point 8. Waterfall plots depict data-point-specific SHAP values. Reading the plots starts at the data-set-averaged output value (*E*[*f*(*x*)], as shown in Table 6). Then each feature’s SHAP value (identified by red arrows if positive and blue arrows if negative) adds to or subtracts from the data-set-averaged output value, giving as the final answer the data-point-specific output value (*f*(*x*)) for a given set of feature values.

On the other hand, hemagglutination had 5 data points that performed well (data points 7′, 8′, 10′, 11′, and 14′). Different features take on different levels of importance depending on the data point, but some trends can be observed overall (Figure 8). First, a cationic monomer percentage of at least 50% is beneficial. The triblock copolymer conformation and DMAEAM monomer, either combined or alone, contributed in a favorable way. A DP of 50 was also favorable. Both models agree with these observations. It is important to compare these with the results expected from the beeswarm plot, which identified DMAEAM as the best monomer but diblock as the best polymer conformation (Table 7). However, from the waterfall plot (Figure 8), diblock copolymers were slightly unfavorable (or had no effect) for the best-performing data points 7′, 8′, and 10′.

**Figure 8:**
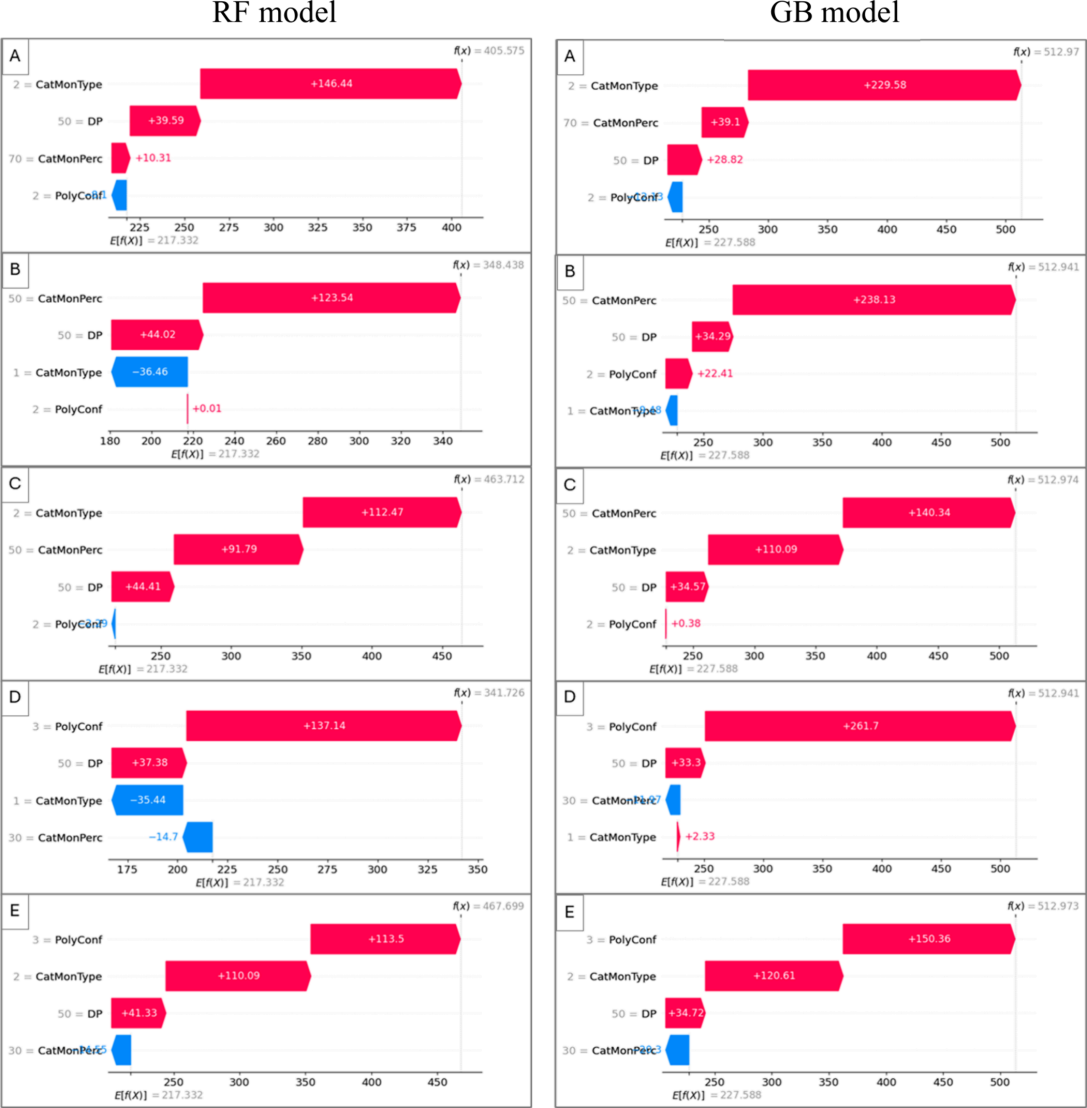
Waterfall plot for best-performing agglutination SAMP designs using the RF model (left) and the GB model (right). (A) data point 7′; (B) data point 8′; (C) data point 10′; (D) data point 11′; (E) data point 14′. Waterfall plots depict data-point-specific SHAP values. Reading the plots starts at the data set-averaged output value (*E*[*f*(*x*)], as shown in Table 6). Then, each feature’s SHAP value (identified by red arrows if positive and blue arrows if negative) adds to or subtracts from the data-set-averaged output value, giving as the final answer the data-point-specific output value (*f*(*x*)) for a given set of feature values.

It should be noted that the most contributing features for the best-performing data points do not always match in type and amount of contribution with the most contributing features from the average SHAP value or FI contribution distributions (Figure 4). The average SHAP value and FI yield data-set-averaged contributions, and so they also take into account data points that did not perform as well. Hence, it may be possible that some of the most effective features to the output contributed in a negative way.

## Discussion

Among the models tested, the tree-ensemble regression methods had the best fit and reproducibility after multiple runs, which was different from a similar study by Kundi et al. that proposed the use of the decision tree classification model with another set of descriptors.^64^ This difference is due to the nature of the datasets used. In the current work, the number of positive cases (class 1) is small, which causes classification models to be irreproducible due to the limited number of class 1 datapoints in the training/test datasets. Here, after running FI and dataset-averaged SHAP analyses for the GB and RF regression models, the features’ importance and their values that most contributed to performance were elucidated for each output as follows (in order of higher to lower contribution): For MIC-*P. aeruginosa* (both strains): the type of cationic monomer (AEAM), cationic ratio (70–100%), chain length (DP100), and polymer conformation (triblock). For MIC-*S. aureus* (both strains): the cationic ratio (70–100%), type of cationic monomer (AEAM), chain length (DP100), and polymer conformation (diblock). For hemagglutination: the type of cationic monomer (DMAEAM), polymer conformation (diblock), chain length (DP100), and cationic ratio (50%).

There are similarities between SAR patterns detected here and our SAR analysis results in a previous work.^44^ The beeswarm plot model feature suggestions align with some of the experimental observations of the best-performing SAMP design features, especially for the polymer conformation, type, and percentage of cationic monomer. However, complete alignment is unexpected, first because the beeswarm plot analysis does not consider any potential feature correlations. Second, insights based on the models may contain feature combinations that have not been experimentally collected, thus not allowing for a direct match but providing additional information. According to the beeswarm plot, the cationic moiety and the cationic ratio are the most contributing features to the potency against *S. aureus* strains, which matches our conclusion based on experimental observations. Interestingly, FI and average SHAP value analyses suggest that DP is the least important feature for activity against *S. aureus* strains, which corroborates the idea that all other features being at their optimal values, the DP value can vary in the 50–100 range without affecting the performance.

This shows the suitability of the descriptors used here as they all contributed to the activity and selectivity of the polymers. The SHAP analysis can be used as a guideline to design future SAMP libraries of similar structures with a higher success rate. For example, utilizing the cationic monomer AEAM in high ratio (>50%) contributes to a high potency and can be used in segmented conformation (diblock or triblock) with DP100. It must be noted that the data set used here is limited and can benefit from more data points that cover a wider range of values.

Furthermore, data-point specific SHAP analysis allowed for the identification of features and feature values that provided the best performance for each output variable. The most influential features, and their corresponding values, for the best-performing data points were for MIC-*P. aeruginosa* (PA14 strain): polymer conformation (triblock) and type of cationic monomer (AEAM); for MIC-*P. aeruginosa* (LESB58 strain): cationic monomer percentage (70%) and cationic monomer type (AEAM); for MIC-*S. aureus* (both strains): cationic monomer percentage (70-100%) and type of cationic monomer (AEAM); and for hemagglutination: type of cationic monomer (DMAEAM), polymer conformation (triblock), and the cationic ratio (50%). It can be noted that the design features that contributed to the best-performant SAMPs do not always match up with the design features that most contribute to overall performance (good or bad) of all SAMPs. This is especially true for MIC PA14 and hemagglutination, and emphasises the added benefit of datapoint-specific SHAP analysis. Additionally, this approach highlights the compromise needed to achieve a SAMP design that achieves good antimicrobial activity while minimising agglutination. For example, DMAEAM is the monomer of choice to minimise agglutination (i.e. increase the concentration at which agglutination occurs), but AEAM is preferable for achieving bacterial inhibition at lower MICs for all strains. There is a potential to utilise the models developed here to perform multi-objective optimisation within the dataset, seeking to find the SAMP design that best leverages antimicrobial activity against agglutination prevention. A preliminary, rough optimisation exercise was made (more information in the Supplementary Information) to identify potential SAMP designs that could accomplish this compromise. One design features percentage of cationic monomer higher than 60% with triblock polymer conformation and AEAM monomer. This design achieves low MICs for LESB58, USA300 and Newman strains (predicted 64-88 μg/ml), relatively low MIC for PA14 (151 μg/ml), and relatively high agglutination concentration (300 μg/ml). Another design features even higher percentages of cationic monomer also with a triblock copolymer but with DMAEM monomer, achieving a good compromise between MIC USA300 and Newman and Agglutination (around 88 μg/ml for the MICs and 516 μg/ml for agglutination). Moderate compromise is achieved with this second design for MIC LESB58 (predicted 128 μg/ml), but poor compromise with MIC PA14 (508 μg/ml). Caution should be exercised as these designs have not been validated experimentally and the optimisation strategy has not been optimised itself. While certain feature importance predictions can be made with the models developed here, a different model can generate completely different patterns. Indeed, Kundi et al.’s work with other descriptors and model recommended alternative designs.^64^ SHAP values in their work recommended increasing the NIPAM ratio over 0.4, maintaining the clogP between 0.5 and −2 and even the omission of AEAM to increase the likelihood of potent polymers.^64^ Hence, it is important to evaluate different models in small, controlled libraries for the most suitable model to be used with a larger data set, and to identify the relevant input values to be used. For example, it should be mentioned that while the work of Kundi et al. suggested that removing AEAM could increase the potency of polymers, in this work, the presence of structurally similar AEAM monomer was one of the key features influencing SAMPs performance. This difference in feature importance prediction could result from the fact that a single bacterial strain is tested (*P. aeruginosa*, PA01) in Kundi et al.’s work^64^ versus the 4 different strains in this work. Even as identified in the present work, MIC outputs differ for the different bacteria, and some respond more to specific SAMP designs than others. Such insight demonstrates that future modelling approaches that seek to combine multiple antimicrobial polymer libraries would have to consider in addition to polymer descriptors, descriptors related to bacterial strain and class (Gram-positive/negative).

## Conclusions

Screening through a library of 23 potential antimicrobial polyacrylamides, their performance was found to be impacted by the following key features: type of cationic monomer, cationic ratio, and DP and polymer architecture. These features were useful parameters in the ML models and could be utilized in future multi-sourced model approaches to predict future candidates. Among the models operated in this pilot study, tree-ensemble regression models, GB and RF, were good fits of the data set and produced consistent FI values across multiple runs and, hence, have great potential in forecasting prospective SAMP candidates that achieve a good compromise between antimicrobial activity and agglutination. SHAP and FI outcomes highlighted the importance of the type and number of cationic moieties on the polymers’ activity. Although the ML predictions in this work were consistent within this data set, it still requires manifold data points to produce informed predictions. This could be achieved by feeding the model presented in this work (regression GB) with the published libraries of SAMPs using the proposed features as descriptors. As highlighted, in addition to polymer descriptors, descriptors for the bacteria strains studied should be considered in multi-library models.

## Supplementary Material



## Notes

More details on fit metrics and predictability for linear regression and classification models; FI values, reproducibility for the classification models and hyperparameter values, and fitting metrics for each run of every model used in this study; description of the tree structures in full; and tables of average SHAP and FI values corresponding to those shown in Figure 4 in the main text (PDF)

## △

School of Chemical, Materials and Biological Engineering, University of Sheffield, Sir Robert Hadfield Building, Mappin Street, Sheffield S1 3JD, U.K
