# Supplementary material for: Potentials of Machine Learning in Predicting Key Features of Synthetic Antimicrobial Polymers
Source: ACS Polym Au. 2026 Apr 27;6(3):821–38. doi: 10.1021/acspolymersau.5c00140 (PMC13261730; doi:10.1021/acspolymersau.5c00140)
Supplement: Supplementary file 1 [file lg5c00140_si_001.pdf]

Supplementary Information

Potentials of Machine Learning in Predicting Key Features of Synthetic Antimicrobial Polymers

Lena Dalal[1], Deborah Barker (first authors)[2], Nicholas Warren[2], Olivier Cayre[2*], Sebastien Perrier[1,3,4*]

[1] Department of Chemistry, University of Warwick, Gibbet Hill Road, Coventry CV4 7AL, U.K.
[2] Department of Chemical and Process Engineering, University of Leeds, Woodhouse, Leeds LS2 9JT, UK
[3] Warwick Medical School, University of Warwick, Coventry CV4 7AL, U.K.
[4] Faculty of Pharmacy and Pharmaceutical Sciences, Monash University, 381 Royal Parade, Parkville, Victoria 3052, Australia

* Corresponding authors: O.J.Cayre@leeds.ac.uk; S.Perrier@warwick.ac.uk

Keywords: Antimicrobial polymers, RAFT, Machine learning, Random forest, Gradient boosting

The fit and predictability of the ML methods

As a first step prior to application of more complex ML models, a simple, linear regression model was fitted. The R^2 values for the models are listed in Table S 1. The linear model could not capture the complexity and non-linearity of the dataset, thus justifying the use of more complex models. The parity plot for the linear models is shown in Figure S 1.

Table S 2 and Table S 3 list the fit for each class for all classification models. The macro-averaged fitting metric values are shown in the main text. Fitting metrics for regression models are shown in Table 4 in the main text. Residual plots for Random Forest and Gradient boosting regression models are shown in Figure S 2.

Table S 1. Goodness of fit metrics for linear regression models.

Output variable	R^2 (training)	R^2 (testing)
MIC PA14	0.38	0.0
MIC LESB58	0.57	-0.37
MIC USA300	0.83	-0.12
MIC Newman	0.79	0.06
Agglutination	0.15	-0.19

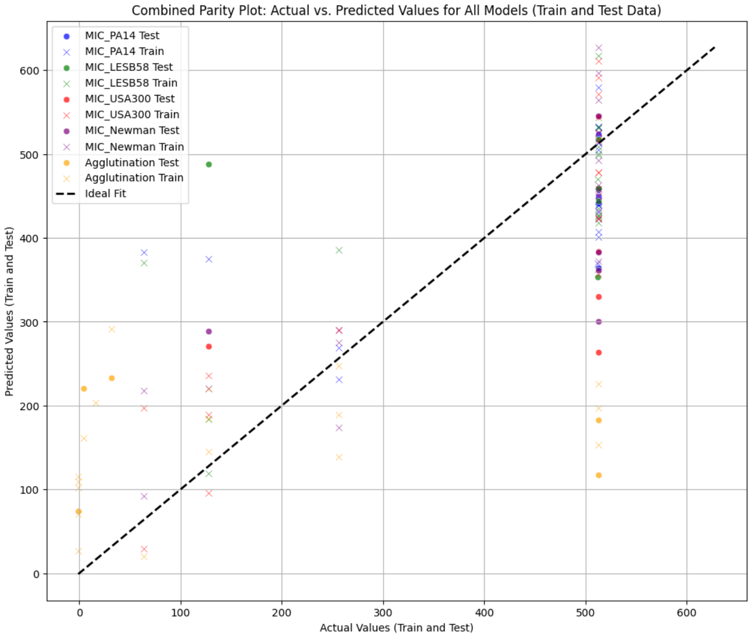

Figure S 1. Parity plot for linear models. The model-predictive output values are plotted against the actual values for both training (crosses) and testing (circles) datasets. The different colours indicate each of the five models tested, one model for each output variable: MIC PA14, MIC LESB58, MIC USA300, MIC Newman, and Agglutination.

Table S 2. Goodness of fit metrics for classification models (class 0).

**Method**	**Output variable**	**Precision***		**Recall***		**F1 score***	
**Decision tree**	*MIC PA14*	1	1	1	1	1	1
	MIC LESB58	1	1	1	0.92	1	0.96
	MIC USA300	1	0.9	1	0.82	1	0.86
	MIC Newman	1	0.89	1	0.73	1	0.8
	Agglutination	1	0.5	1	0.33	1	0.4
**Random forest**	*MIC PA14*	1	1	1	1	1	1
	MIC LESB58	1	1	1	1	1	1

	MIC USA300	1	0.92	1	1	1	0.96
	MIC Newman	1	0.92	1	1	1	0.96
	Agglutination	1	0.5	1	1	1	0.67
**Gradient boosting**	*MIC PA14*	1	1	1	1	1	1
	MIC LESB58	1	1	1	0.5	1	0.86
	MIC USA300	1	0.91	1	0.91	1	0.91
	MIC Newman	1	1	1	0.91	1	0.95
	Agglutination	1	0.33	1	0.67	1	0.44

*The first value is for the training dataset, and the second, for the validation dataset. The macro-averaged precision, recall and F1 score for each class are shown in the main text.

Table S 3. Goodness of fit metrics for classification models (class 1)

**Method**	**Output variable**	**Precision***		**Recall***		**F1 score***	
**Decision tree**	*MIC PA14*	1	N/A	1	N/A	1	N/A
	MIC LESB58	1	0	1	0	1	0
	MIC USA300	1	0	1	0	1	0
	MIC Newman	1	0	1	0	1	0
	Agglutination	1	0.71	1	0.83	1	0.77
**Random forest**	*MIC PA14*	1	N/A	1	N/A	1	N/A
	MIC LESB58	1	N/A	1	N/A	1	N/A
	MIC USA300	1	0	1	0	1	0
	MIC Newman	1	0	1	0	1	0
	Agglutination	1	1	1	0.71	1	0.83
**Gradient boosting**	*MIC PA14*	1	N/A	1	N/A	1	N/A
	MIC LESB58	1	0	1	0	1	0
	MIC USA300	1	0	1	0	1	0
	MIC Newman	1	0.5	1	1	1	0.67
	Agglutination	1	0.67	1	0.33	1	0.44

*The first value is for the training dataset, and the second, for the validation dataset. The macro-averaged precision, recall and F1 score for each class are shown in the main text. N/A = not available, that is this class was not present in the testing dataset (and no testing datapoint was miss-assigned to this class).

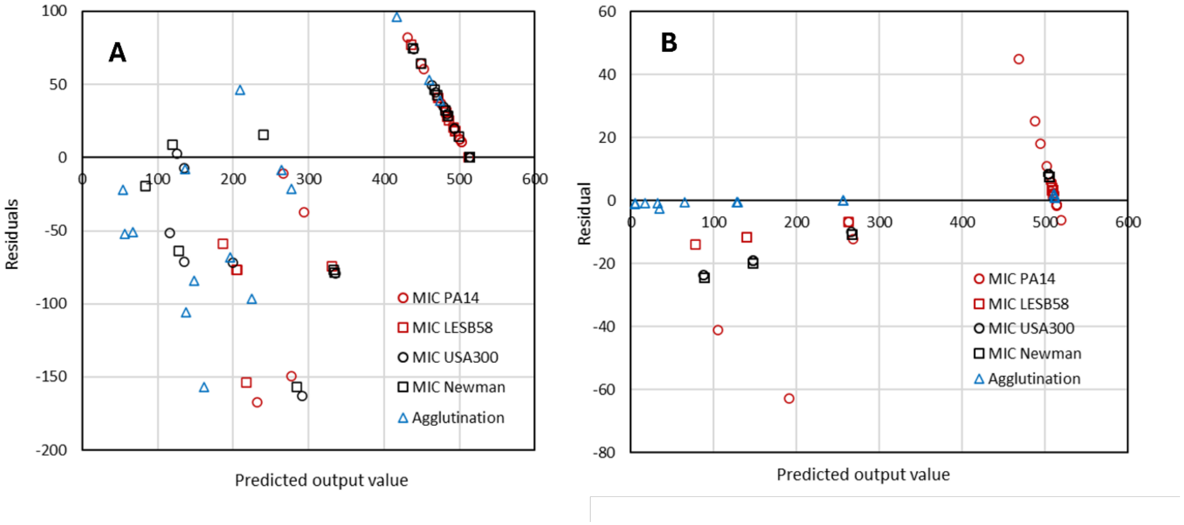

Figure S 2. Residual plots for Random forest (A) and Gradient boosting (B) regression models. The residual is the error between the predicted and actual value (positive if actual value is larger than the predicted value, and negative if otherwise). Residuals for models modelling all five output variables (MIC PA14, MIC LESB58, MIC USA300, MIC Newman and Agglutination) are shown in the graphs.

For MIC PA14 and MIC LESB58, only one datapoint in each belonged to class 1. This datapoint was always in the training dataset, which resulted in some metrics in the testing dataset set having N/A values or 0 (Table S 3). In this case, N/A means that class 1 was not present in the testing dataset (and no testing datapoint was miss-assigned to class 1), while a value of 0 means that some testing datapoints (all of which truly belonging to class 0) were incorrectly assigned to class 1. For better understanding of this, Figure S 3Figure S 3 shows the confusion matrices for the Gradient boosting classification models of MIC PA14 and MIC LESB58, as an example, and equations for precision, recall and F1 score calculations are shown below. For MIC USA300 and MIC Newman, two datapoints existed in class 1, and so one was always in the training dataset, and the other in the testing dataset, so this issue did not occur. Agglutination had an almost even split between classes. When N/A shows in a metric, the macro-averaged result does not include this value, and so the macro-averaged metric may give a false sense of goodness of fit.

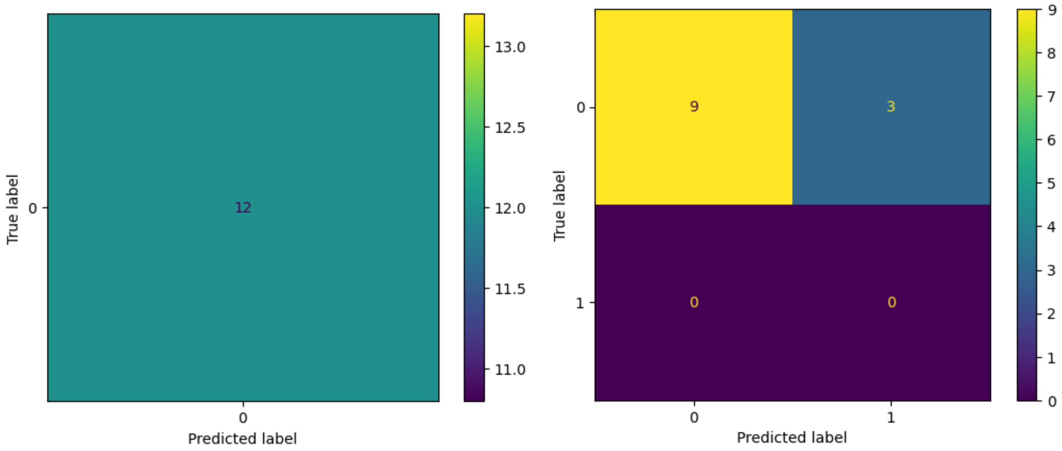

Figure S 3. Testing dataset confusion matrices for MIC PA14 (left) and MIC LESB58 (right) for gradient boosting classification model. In both cases, no datapoint from class 1 was present in the testing dataset. For MIC PA14, all datapoints were correctly assigned to class 0, meaning, no fitting metric information is available for class 1, while for MIC LESB58, some class 0 datapoints were incorrectly assigned to class 1, meaning metrics for this class could be calculated and equal 0.

$$\text{Precision} = \frac{\text{true positive}}{\text{true positive} + \text{false positive}} = \frac{\text{true class 0 (or class 1)}}{\text{true class 0 (or class 1)} + \text{false class 0 (or class 1)}}$$

$$\text{Recall} = \frac{\text{true positive}}{\text{true positive} + \text{false negative}} = \frac{\text{true class 0 (or class 1)}}{\text{true class 0 (or class 1)} + \text{false class 1 (or class 0)}}$$

$$\text{F1 score} = \frac{2}{\frac{1}{\text{Precision}} + \frac{1}{\text{Recall}}}$$

Reproducibility of feature importance

Table S 4 lists the average feature importance and standard error for each output variable and classification model.

Table S 4. Average feature importance, standard deviation and error for classification models

Output variable	Feature	Decision tree			Random forest			Gradient boosting		
		Avg	Stdev	Stder (%)	Avg	Stdev	Stder (%)	Avg	Stdev	Stder (%)
MIC-PA14	*DP*	0.00	0.00	0	0.09	0.05	25	0.04	0.08	100
	CatMonPerc	0.11	0.25	100	0.09	0.05	25	0.18	0.32	78
	PolyConf	0.73	0.39	24	0.53	0.15	13	0.59	0.37	28
	CatMonType	0.17	0.25	67	0.29	0.18	28	0.20	0.23	51
MIC-LESB58	*DP*	0.22	0.30	61	0.22	0.18	35	0.33	0.30	41
	CatMonPerc	0.63	0.32	22	0.47	0.15	14	0.37	0.31	37
	PolyConf	0.00	0.00	0	0.09	0.05	26	0.00	0.00	0
	CatMonType	0.15	0.10	30	0.22	0.09	17	0.30	0.10	15
MIC-USA300	*DP*	0.00	0.00	0	0.03	0.04	62	0.39	0.24	27
	CatMonPerc	0.44	0.29	30	0.42	0.13	14	0.16	0.11	30
	PolyConf	0.20	0.45	100	0.32	0.27	38	0.20	0.45	100
	CatMonType	0.37	0.26	32	0.23	0.18	34	0.25	0.16	28
MIC-Newman	*DP*	0.22	0.30	61	0.01	0.01	48	0.00	0.00	0
	CatMonPerc	0.67	0.30	20	0.38	0.09	10	0.12	0.19	69
	PolyConf	0.00	0.00	0	0.33	0.25	34	0.77	0.34	20
	CatMonType	0.11	0.25	100	0.27	0.22	37	0.11	0.25	100
C_H	*DP*	0.25	0.42	75	0.15	0.05	16	0.19	0.20	46
	CatMonPerc	0.25	0.25	45	0.38	0.16	19	0.39	0.10	11
	PolyConf	0.24	0.28	53	0.13	0.06	21	0.02	0.04	100
	CatMonType	0.26	0.17	30	0.35	0.09	12	0.39	0.28	32

The Tables that follow (Table S 5 to Table S 10) present the feature importance data for all runs used to calculate the average values.

Table S 5. Feature importance values for each run (Decision tree regression)

		Run 1	Run 2	Run 3	Run 4	Run 5
**MIC PA14**	DP	0.24	0.21	0.18	0.21	0.20
	CatMonPerc	0.14	0.00	0.00	0.36	0.29
	PolyConf	0.39	0.68	0.64	0.05	0.31
	CatMonType	0.24	0.11	0.17	0.38	0.19
	check	1.00	1.00	1.00	1.00	1.00
**MIC LESB58**	DP	0.32	0.47	0.37	0.01	0.26
	CatMonPerc	0.38	0.09	0.30	0.25	0.24
	PolyConf	0.15	0.04	0.17	0.00	0.00
	CatMonType	0.15	0.40	0.15	0.75	0.50
	check	1.00	1.00	1.00	1.00	1.00
**MIC USA300**	DP	0.10	0.19	0.08	0.08	0.02
	CatMonPerc	0.68	0.68	0.60	0.72	0.47
	PolyConf	0.21	0.02	0.15	0.14	0.32
	CatMonType	0.02	0.12	0.17	0.06	0.19
	check	1.00	1.00	1.00	1.00	1.00
**MIC Newman**	DP	0.21	0.07	0.01	0.00	0.11
	CatMonPerc	0.54	0.60	0.40	0.56	0.38
	PolyConf	0.09	0.13	0.20	0.18	0.18
	CatMonType	0.16	0.20	0.39	0.26	0.32
	check	1.00	1.00	1.00	1.00	1.00
**Agglutination**	DP	0.10	0.00	0.00	0.23	0.00
	CatMonPerc	0.34	0.45	0.38	0.29	0.52
	PolyConf	0.27	0.39	0.21	0.06	0.21
	CatMonType	0.29	0.16	0.41	0.42	0.27
	check	1.00	1.00	1.00	1.00	1.00

Table S 6. Feature importance values for each run (RF regression)

		Run 1	Run 2	Run 3	Run 4	Run 5
**MIC PA14**	DP	0.28	0.35	0.46	0.43	0.41
	CatMonPerc	0.16	0.21	0.18	0.09	0.16
	PolyConf	0.25	0.25	0.15	0.23	0.19
	CatMonType	0.31	0.19	0.22	0.24	0.24
	check	1.00	1.00	1.00	1.00	1.00
**MIC LESB58**	DP	0.31	0.37	0.32	0.38	0.27
	CatMonPerc	0.26	0.26	0.23	0.22	0.29
	PolyConf	0.10	0.06	0.16	0.08	0.10
	CatMonType	0.33	0.31	0.29	0.32	0.35
	check	1.00	1.00	1.00	1.00	1.00
**MIC USA300**	DP	0.11	0.11	0.11	0.13	0.15
	CatMonPerc	0.61	0.60	0.60	0.60	0.59
	PolyConf	0.10	0.13	0.13	0.14	0.13
	CatMonType	0.18	0.16	0.15	0.13	0.13
	check	1.00	1.00	1.00	1.00	1.00
**MIC Newman**	DP	0.13	0.11	0.12	0.08	0.15
	CatMonPerc	0.54	0.57	0.52	0.58	0.53
	PolyConf	0.14	0.14	0.14	0.15	0.12
	CatMonType	0.19	0.18	0.23	0.18	0.20
	check	1.00	1.00	1.00	1.00	1.00
**Agglutination**	DP	0.12	0.17	0.16	0.12	0.11
	CatMonPerc	0.20	0.27	0.27	0.29	0.26
	PolyConf	0.30	0.24	0.23	0.25	0.26
	CatMonType	0.38	0.32	0.35	0.34	0.37
	check	1.00	1.00	1.00	1.00	1.00

Table S 7. Feature importance values for each run (GB regression)

		Run 1	Run 2	Run 3	Run 4	Run 5
**MIC PA14**	DP	0.15	0.15	0.20	0.15	0.15
	CatMonPerc	0.26	0.26	0.25	0.26	0.26
	PolyConf	0.33	0.33	0.30	0.33	0.33
	CatMonType	0.26	0.26	0.25	0.26	0.26
	check	1.00	1.00	1.00	1.00	1.00
**MIC LESB58**	DP	0.36	0.36	0.36	0.36	0.36
	CatMonPerc	0.37	0.37	0.37	0.37	0.37
	PolyConf	0.10	0.10	0.10	0.10	0.10
	CatMonType	0.17	0.17	0.17	0.17	0.17
	check	1.00	1.00	1.00	1.00	1.00
**MIC USA300**	DP	0.07	0.07	0.07	0.07	0.07
	CatMonPerc	0.52	0.52	0.52	0.52	0.52
	PolyConf	0.14	0.14	0.14	0.14	0.14
	CatMonType	0.26	0.26	0.26	0.26	0.26
	check	1.00	1.00	1.00	1.00	1.00
**MIC Newman**	DP	0.09	0.09	0.09	0.09	0.09
	CatMonPerc	0.57	0.57	0.57	0.57	0.57
	PolyConf	0.17	0.17	0.17	0.17	0.17
	CatMonType	0.18	0.18	0.18	0.18	0.18
	check	1.00	1.00	1.00	1.00	1.00
**Agglutination**	DP	0.18	0.18	0.18	0.18	0.18
	CatMonPerc	0.23	0.22	0.22	0.22	0.22
	PolyConf	0.20	0.20	0.20	0.20	0.20
	CatMonType	0.39	0.39	0.40	0.40	0.40
	check	1.00	1.00	1.00	1.00	1.00

Table S 8. Feature importance values for each run (Decision tree classification)

		Run 1	Run 2	Run 3	Run 4	Run 5
**MIC PA14**	DP	0.00	0.00	0.00	0.00	0.00
	CatMonPerc	0.00	0.00	0.55	0.00	0.00
	PolyConf	0.45	1.00	0.18	1.00	1.00
	CatMonType	0.55	0.00	0.28	0.00	0.00
	check	1.00	1.00	1.00	1.00	1.00
**MIC LESB58**	DP	0.00	0.55	0.00	0.00	0.55
	CatMonPerc	1.00	0.28	0.73	0.83	0.33
	PolyConf	0.00	0.00	0.00	0.00	0.00
	CatMonType	0.00	0.18	0.27	0.18	0.12
	check	1.00	1.00	1.00	1.00	1.00
**MIC USA300**	DP	0.00	0.00	0.00	0.00	0.00
	CatMonPerc	0.83	0.45	0.45	0.00	0.45
	PolyConf	0.00	0.00	0.00	1.00	0.00
	CatMonType	0.18	0.55	0.55	0.00	0.55
	check	1.00	1.00	1.00	1.00	1.00
**MIC Newman**	DP	0.55	0.00	0.00	0.55	0.00
	CatMonPerc	0.45	0.45	1.00	0.45	1.00
	PolyConf	0.00	0.00	0.00	0.00	0.00
	CatMonType	0.00	0.55	0.00	0.00	0.00
	check	1.00	1.00	1.00	1.00	1.00
**Agglutination**	DP	0.09	0.18	0.00	1.00	0.00
	CatMonPerc	0.27	0.56	0.00	0.00	0.44
	PolyConf	0.18	0.00	0.67	0.00	0.33
	CatMonType	0.47	0.27	0.33	0.00	0.22
	check	1.00	1.00	1.00	1.00	1.00

Table S 9. Feature importance values for each run (RF classification)

		Run 1	Run 2	Run 3	Run 4	Run 5
**MIC PA14**	DP	0.07	0.02	0.11	0.12	0.15
	CatMonPerc	0.08	0.11	0.03	0.07	0.16
	PolyConf	0.28	0.65	0.59	0.50	0.62
	CatMonType	0.58	0.23	0.27	0.31	0.07
	check	1.00	1.00	1.00	1.00	1.00
**MIC LESB58**	DP	0.33	0.04	0.41	0.03	0.29
	CatMonPerc	0.31	0.60	0.30	0.59	0.52
	PolyConf	0.06	0.16	0.13	0.04	0.05
	CatMonType	0.29	0.21	0.15	0.33	0.13
	check	1.00	1.00	1.00	1.00	1.00
**MIC USA300**	DP	0.00	0.08	0.00	0.06	0.00
	CatMonPerc	0.37	0.44	0.49	0.57	0.22
	PolyConf	0.27	0.04	0.49	0.11	0.70
	CatMonType	0.36	0.44	0.02	0.26	0.08
	check	1.00	1.00	1.00	1.00	1.00
**MIC Newman**	DP	0.02	0.01	0.00	0.04	0.00
	CatMonPerc	0.44	0.41	0.38	0.44	0.23
	PolyConf	0.10	0.55	0.58	0.03	0.41
	CatMonType	0.44	0.03	0.04	0.49	0.35
	check	1.00	1.00	1.00	1.00	1.00
**Agglutination**	DP	0.06	0.17	0.19	0.15	0.16
	CatMonPerc	0.59	0.23	0.50	0.34	0.24
	PolyConf	0.10	0.21	0.05	0.14	0.13
	CatMonType	0.25	0.39	0.26	0.36	0.47
	check	1.00	1.00	1.00	1.00	1.00

Table S 10. Feature importance values for each run (GB classification)

		Run 1	Run 2	Run 3	Run 4	Run 5
**MIC PA14**	DP	0.18	0.00	0.00	0.00	0.00
	CatMonPerc	0.00	0.00	0.17	0.73	0.00
	PolyConf	0.82	0.45	0.83	0.00	0.83
	CatMonType	0.00	0.55	0.00	0.27	0.18
	check	1.00	1.00	1.00	1.00	1.00
**MIC LESB58**	DP	0.55	0.55	0.00	0.55	0.00
	CatMonPerc	0.18	0.12	0.55	0.18	0.83
	PolyConf	0.00	0.00	0.00	0.00	0.00
	CatMonType	0.28	0.33	0.45	0.28	0.17
	check	1.00	1.00	1.00	1.00	1.00
**MIC USA300**	DP	0.55	0.55	0.00	0.32	0.55
	CatMonPerc	0.24	0.12	0.00	0.27	0.18
	PolyConf	0.00	0.00	1.00	0.00	0.00
	CatMonType	0.21	0.33	0.00	0.41	0.28
	check	1.00	1.00	1.00	1.00	1.00
**MIC Newman**	DP	0.00	0.00	0.00	0.00	0.00
	CatMonPerc	0.43	0.00	0.00	0.00	0.18
	PolyConf	0.57	1.00	1.00	1.00	0.27
	CatMonType	0.00	0.00	0.00	0.00	0.55
	check	1.00	1.00	1.00	1.00	1.00
**Agglutination**	DP	0.00	0.24	0.26	0.00	0.48
	CatMonPerc	0.43	0.43	0.33	0.27	0.52
	PolyConf	0.00	0.00	0.08	0.00	0.00
	CatMonType	0.57	0.33	0.33	0.73	0.00
	check	1.00	1.00	1.00	1.00	1.00

The hyperparameter values and fitting metrics for each model run are shown in the tables below (Table S 11 to Table S 16).

Table S 11. Hyperparameter values and fitting metrics for each run (Decision tree regression)

		**Run 1**	**Run 2**	**Run 3**	**Run 4**	**Run 5**
**MIC PA14**	R^2	1	1	1	1	1
	n_estimators	1	1	1	1	1
	max_depth	13	17	4	13	8
**MIC LESB58**	R^2	1	1	1	1	1
	n_estimators	1	1	1	1	1
	max_depth	19	18	8	10	7
**MIC USA300**	R^2	1	1	1	1	1
	n_estimators	1	1	1	1	1
	max_depth	18	6	6	14	11
**MIC Newman**	R^2	1	1	1	1	1
	n_estimators	1	1	1	1	1
	max_depth	9	6	18	14	10
**Agglutination**	R^2	1	1	1	1	1
	n_estimators	1	1	1	1	1
	max_depth	18	10	8	13	14

R^2 for validation dataset.

Table S 12. Hyperparameter values and fitting metrics for each run (RF regression)

		Run 1	Run 2	Run 3	Run 4	Run 5
**MIC PA14**	*R^2	0.833	0.796	0.777	0.844	0.798
	n_estimators	31	29	31	35	45
	max_depth	14	6	9	13	7
**MIC LESB58**	R^2	0.941	0.910	0.937	0.935	0.926
	n_estimators	99	51	49	35	37
	max_depth	10	4	19	5	15
**MIC USA300**	R^2	0.903	0.921	0.903	0.897	0.895
	n_estimators	74	51	75	74	37
	max_depth	16	19	6	17	16
**MIC Newman**	R^2	0.877	0.864	0.871	0.864	0.873
	n_estimators	71	70	30	31	29
	max_depth	18	11	6	18	9
**Agglutination**	R^2	0.818	0.841	0.808	0.804	0.851
	n_estimators	41	30	27	43	32
	max_depth	5	8	7	13	16

*R^2 for whole dataset (cross-validation used).

Table S 13. Hyperparameter values and fitting metrics for each run (GB regression)

		Run 1	Run 2	Run 3	Run 4	Run 5
**MIC PA14**	R^2	0.995	0.995	0.973	0.995	0.995
	n_estimators	25	25	26	25	25
	max_depth	8	7	5	17	7
**MIC LESB58**	R^2	1.000	0.996	0.999	0.999	0.998
	n_estimators	50	26	36	35	30
	max_depth	8	18	18	14	9
**MIC USA300**	R^2	0.996	0.995	0.997	0.995	0.996
	n_estimators	26	25	27	25	26
	max_depth	12	6	6	15	14
**MIC Newman**	R^2	0.995	0.996	0.995	0.995	0.997
	n_estimators	25	26	25	25	27
	max_depth	14	19	8	18	10
**Agglutination**	R^2	1.000	1.000	1.000	1.000	1.000
	n_estimators	60	69	72	98	67
	max_depth	5	5	5	5	5

R^2 for whole dataset (cross-validation used).

Table S 14. Hyperparameter values and fitting metrics for each run (Decision tree classification)

		Run 1	Run 2	Run 3	Run 4	Run 5
**MIC PA14**	macro-avg precision	0.5	0.5	0.5	0.5	0.5
	macro-avg recall	0.46	0.42	0.42	0.42	0.42
	macro-avg F1	0.48	0.45	0.45	0.45	0.45
	n_estimators	1	1	1	1	1
	max_depth	5	10	12	12	6
**MIC LESB58**	macro-avg precision	0.5	1	0.5	0.5	1
	macro-avg recall	0.38	1	0.46	0.46	1
	macro-avg F1	0.43	1	0.48	0.48	1
	n_estimators	1	1	1	1	1
	max_depth	8	10	12	19	14
**MIC USA300**	macro-avg precision	0.45	0.45	0.45	0.45	0.46
	macro-avg recall	0.45	0.45	0.45	0.45	0.5
	macro-avg F1	0.45	0.45	0.45	0.45	0.48
	n_estimators	1	1	1	1	1
	max_depth	19	15	14	18	10
**MIC Newman**	macro-avg precision	0.45	0.46	0.45	0.6	0.45
	macro-avg recall	0.41	0.5	0.41	0.82	0.41
	macro-avg F1	0.43	0.48	0.43	0.56	0.43
	n_estimators	1	1	1	1	1
	max_depth	7	13	5	17	6
**Agglutination**	macro-avg precision	0.65	0.61	0.67	0.54	0.28
	macro-avg recall	0.67	0.58	0.65	0.53	0.5
	macro-avg F1	0.65	0.58	0.65	0.5	0.36
	n_estimators	1	1	1	1	1
	max_depth	6	12	15	17	14

Table S 15. Hyperparameter values and fitting metrics for each run (RF classification)

		Run 1	Run 2	Run 3	Run 4	Run 5
**MIC PA14**	macro-avg precision	0.5	0.5	1	1	1
	macro-avg recall	0.42	0.46	1	1	1
	macro-avg F1	0.45	0.48	1	1	1
	n_estimators	32	76	40	80	58
	max_depth	13	4	18	9	16
**MIC LESB58**	macro-avg precision	1	0.5	1	0.5	1
	macro-avg recall	1	0.46	1	0.46	1
	macro-avg F1	1	0.48	1	0.48	1
	n_estimators	30	76	38	59	72
	max_depth	16	6	9	13	8
**MIC USA300**	macro-avg precision	0.46	0.45	0.45	0.46	0.45
	macro-avg recall	0.5	0.45	0.45	0.5	0.45
	macro-avg F1	0.48	0.45	0.45	0.48	0.45
	n_estimators	95	90	94	48	54
	max_depth	19	7	14	12	7
**MIC Newman**	macro-avg precision	0.45	0.45	0.45	0.46	0.46
	macro-avg recall	0.45	0.45	0.45	0.5	0.5
	macro-avg F1	0.45	0.45	0.45	0.48	0.48
	n_estimators	87	39	33	58	74
	max_depth	8	16	7	10	16
**Agglutination**	macro-avg precision	0.75	0.28	0.28	0.93	0.88
	macro-avg recall	0.75	0.5	0.5	0.83	0.67
	macro-avg F1	0.75	0.36	0.36	0.86	0.68
	n_estimators	26	37	87	86	50
	max_depth	14	15	19	8	13

Table S 16. Hyperparameter values and fitting metrics for each run (GB classification)

		**Run 1**	**Run 2**	**Run 3**	**Run 4**	**Run 5**
**MIC PA14**	macro-avg precision	0.5	0.5	0.5	0.5	0.5
	macro-avg recall	0.42	0.33	0.42	0.29	0.25
	macro-avg F1	0.45	0.4	0.45	0.37	0.33
	n_estimators	42	59	56	47	71
	max_depth	13	7	13	17	8
**MIC LESB58**	macro-avg precision	1	1	0.5	1	0.5
	macro-avg recall	1	1	0.42	1	0.46
	macro-avg F1	1	1	0.45	1	0.48
	n_estimators	53	55	74	73	97
	max_depth	5	7	17	19	18
**MIC USA300**	macro-avg precision	0.46	0.46	0.45	0.46	0.75
	macro-avg recall	0.5	0.5	0.45	0.5	0.95
	macro-avg F1	0.48	0.48	0.45	0.48	0.81
	n_estimators	55	77	35	99	44
	max_depth	8	10	5	6	7
**MIC Newman**	macro-avg precision	0.45	0.45	0.45	0.45	0.46
	macro-avg recall	0.45	0.45	0.45	0.45	0.5
	macro-avg F1	0.45	0.45	0.45	0.45	0.48
	n_estimators	61	42	98	85	48
	max_depth	9	12	17	13	8
**Agglutination**	macro-avg precision	0.75	0.67	0.81	0.35	0.67
	macro-avg recall	0.6	0.65	0.62	0.33	0.65
	macro-avg F1	0.5	0.65	0.58	0.33	0.65
	n_estimators	73	95	44	25	61
	max_depth	11	8	9	9	14

Additional spider plots for decision tree and GB regression models, and all classification models are shown in Figure S 4 to Figure S 8. (the plot for regression RF is in the main text).

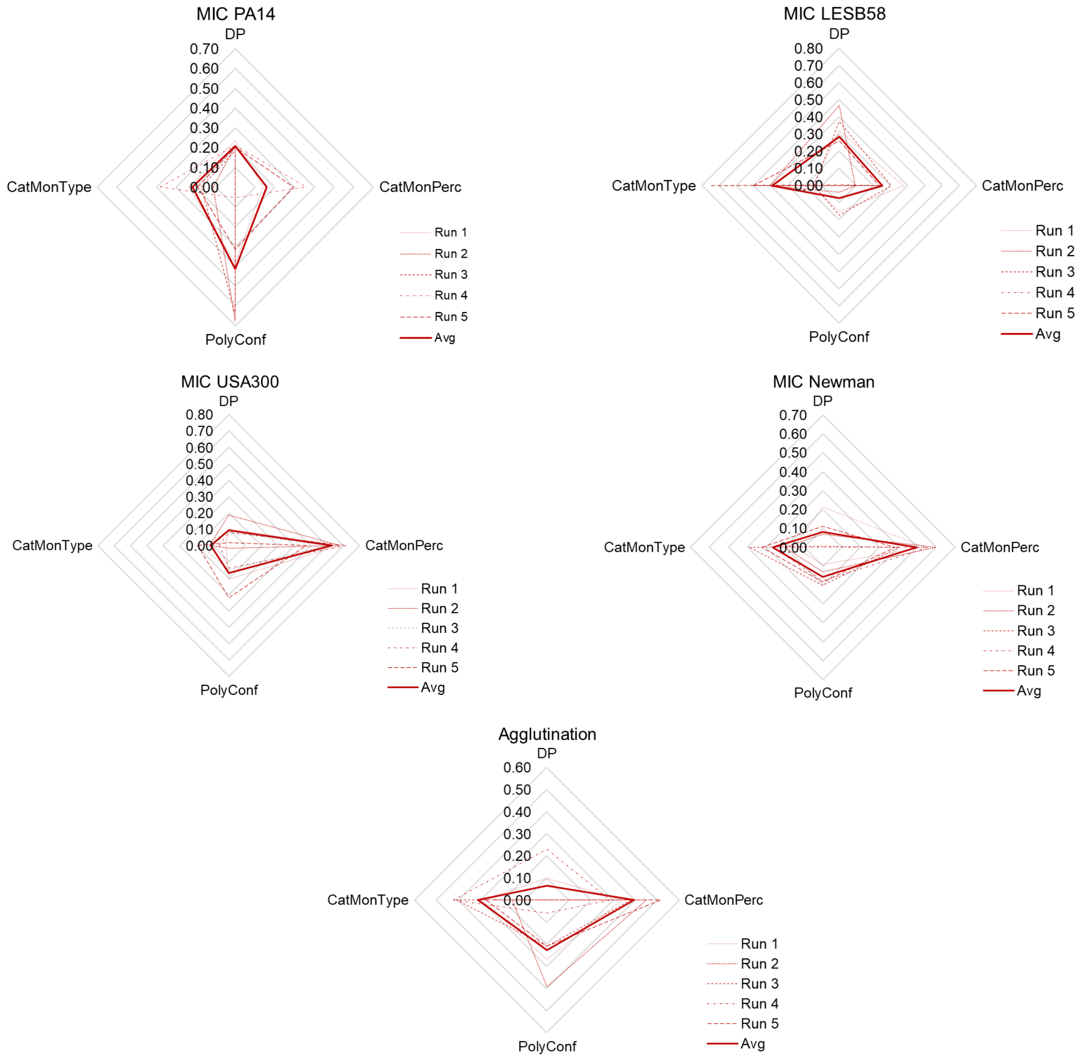

Figure S 4. Reproducibility test of feature importance in 5 different runs for regression decision tree models.

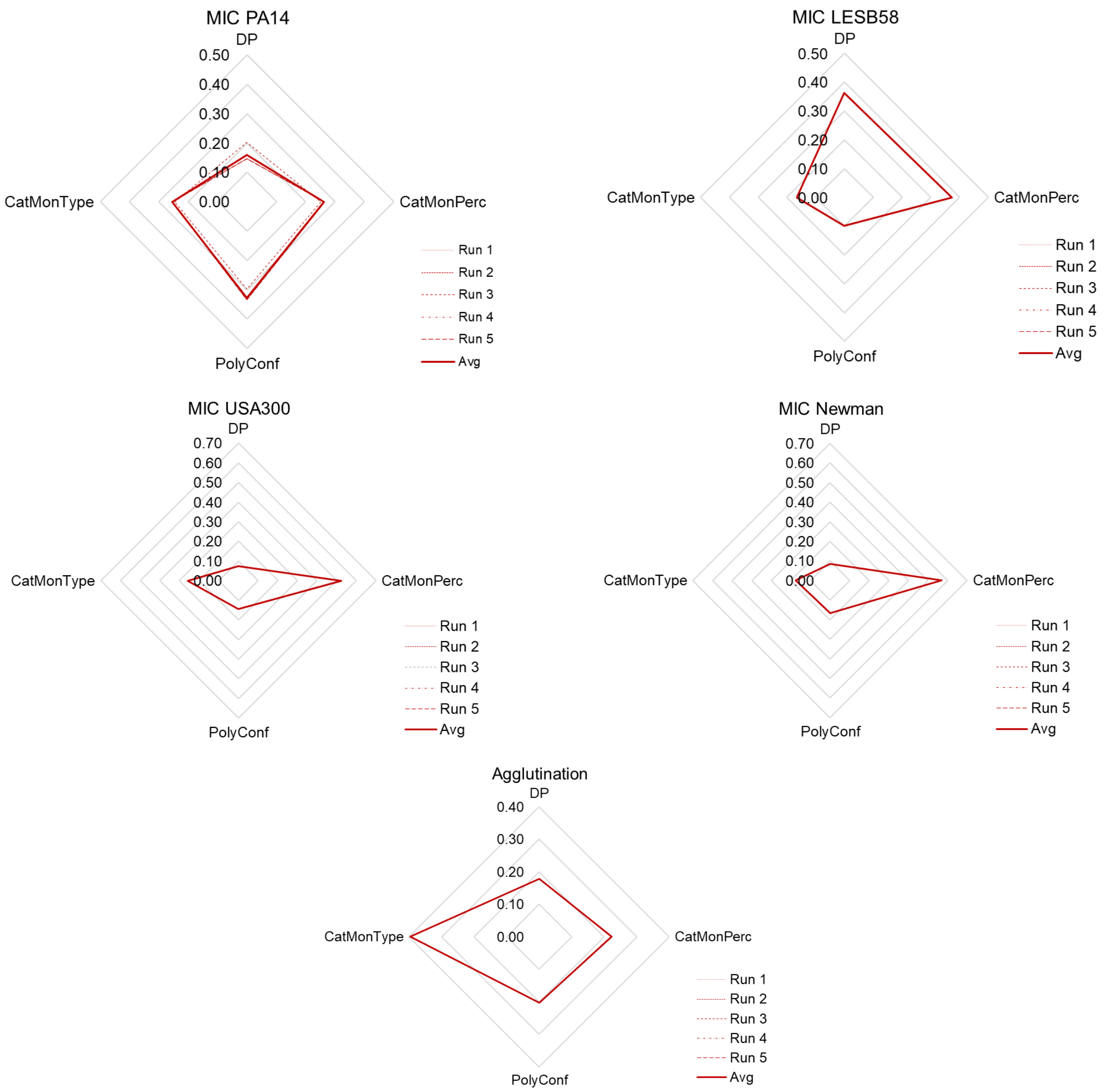

Figure S 5. Reproducibility test of feature importance in 5 different runs for regression gradient boosting models.

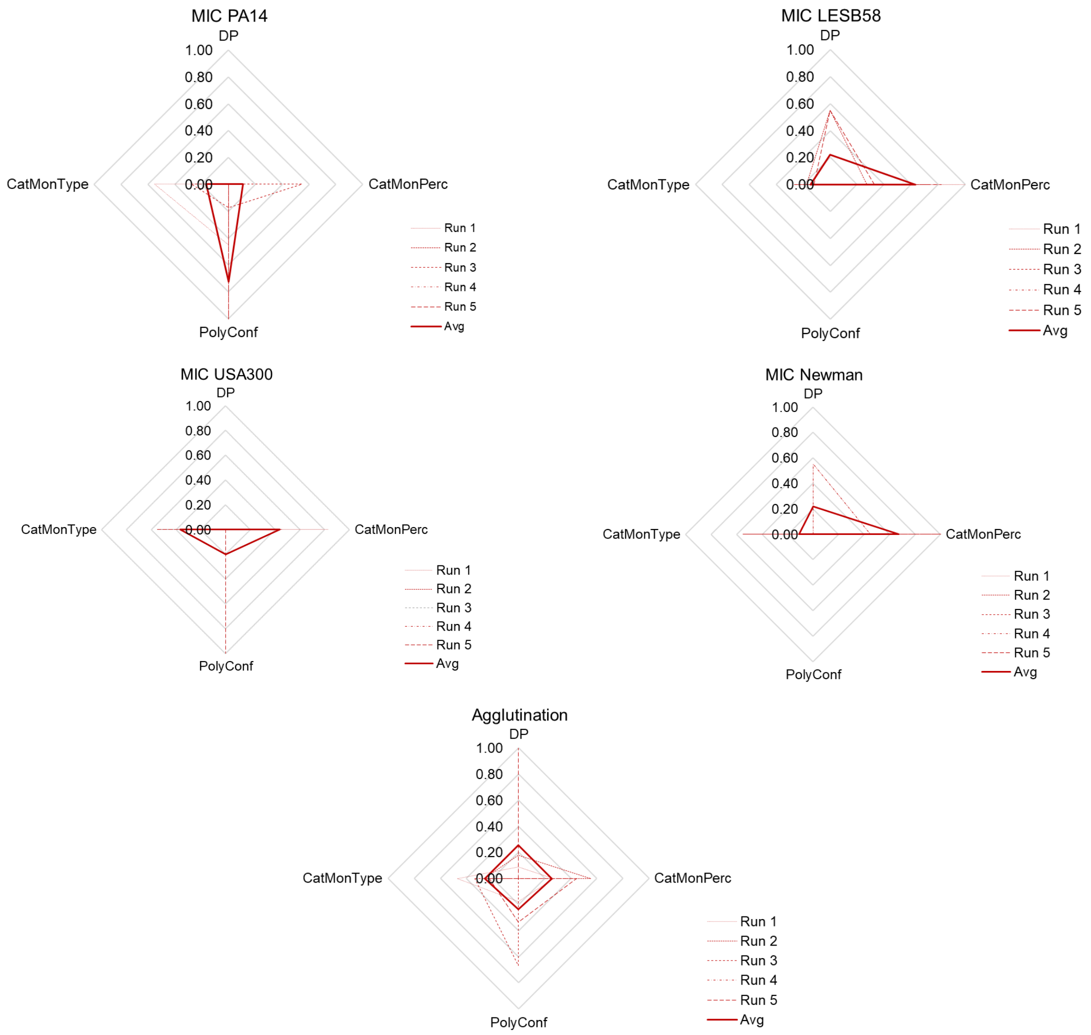

Figure S 6. Reproducibility test of feature importance in 5 different runs classification decision tree models.

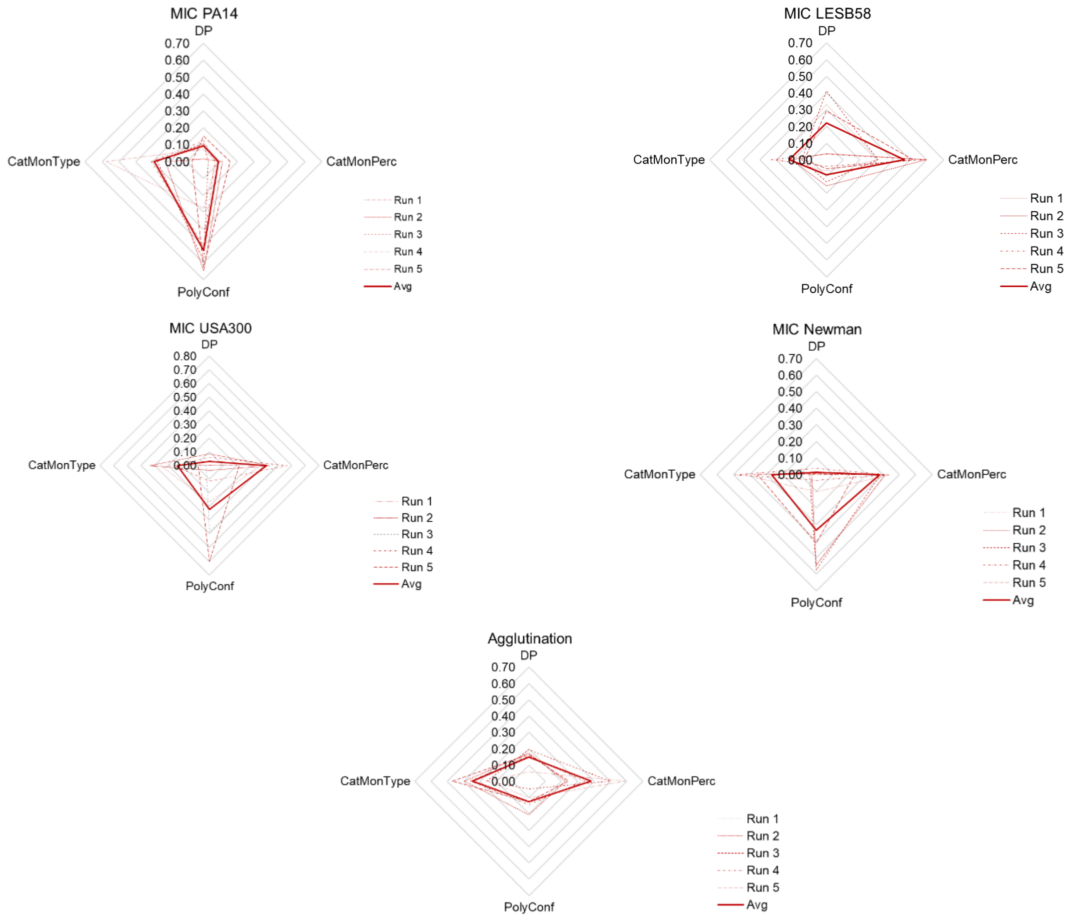

Figure S 7.Reproducibility test of feature importance in 5 different runs classification random forest models.

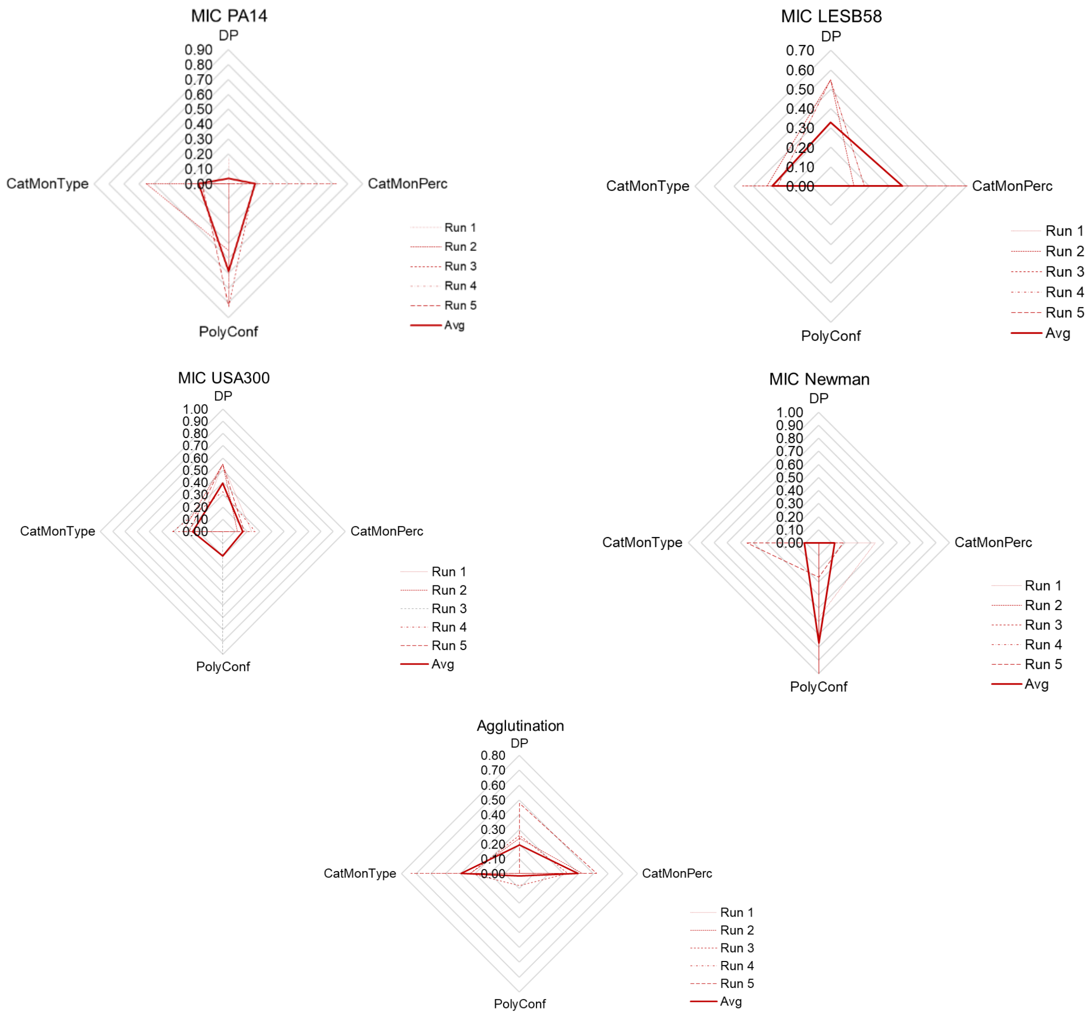

Figure S 8. Reproducibility test of feature importance in 5 different runs classification gradient boosting models.

Tree structure consistency

The structure of trees (single decision tree or the first and last trees in gradient boosting) were analysed for different runs and compared to check for consistency. For random forests, as the average output for every tree in the ensemble is bagged together to give the final output, the structure of each tree is less important, and so tree structure consistency was not verified for RF models.

For decision tree models, the structure of the single fitted tree was compared over different runs. For the regression decision tree models, some tree structure examples are shown in the figures that follow. Figure S 9 shows tree structures for runs 1 and 3 of the MIC PA14 model. As seen, not only are the node structures and splitting different, but the features over which nodes at the same depth split are distinct. Similar observations were made for MIC LESB58 (Figure S 10). For MIC USA300 and MIC Newman, tree structures were slightly different but presented many shared node-splitting and feature selection. This is illustrated for MIC USA300 in Figure S 11. These more similar tree structures for MIC USA300 and MIC Newman explain why these two outputs had less dissimilar feature importance distributions than MIC PA14 or MIC LESB59 for example (see spider plots in Figure S 4).

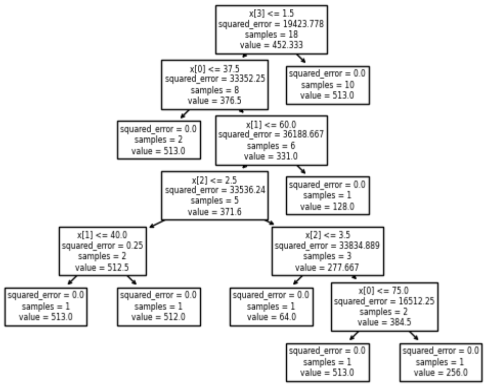

Figure S 9. Different tree structures for MIC PA14 regression decision tree models. Left: tree fitted in run 1. Right: tree fitted in run 3. X[0] = DP, x[1] = CatMonPerc, x[2] = PolyConf, and x[3] = CatMonType.

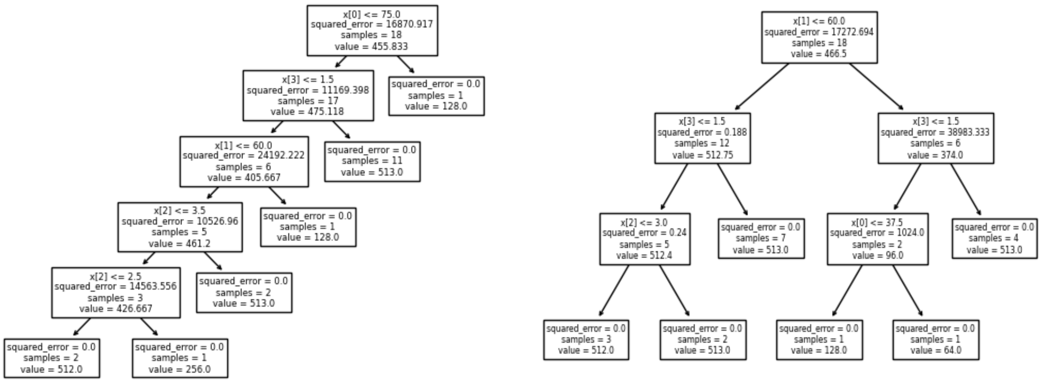

Figure S 10. Different tree structures for MIC LESB58 regression decision tree models. Left: tree fitted in run 2. Right: tree fitted in run 3. X[0] = DP, x[1] = CatMonPerc, x[2] = PolyConf, and x[3] = CatMonType.

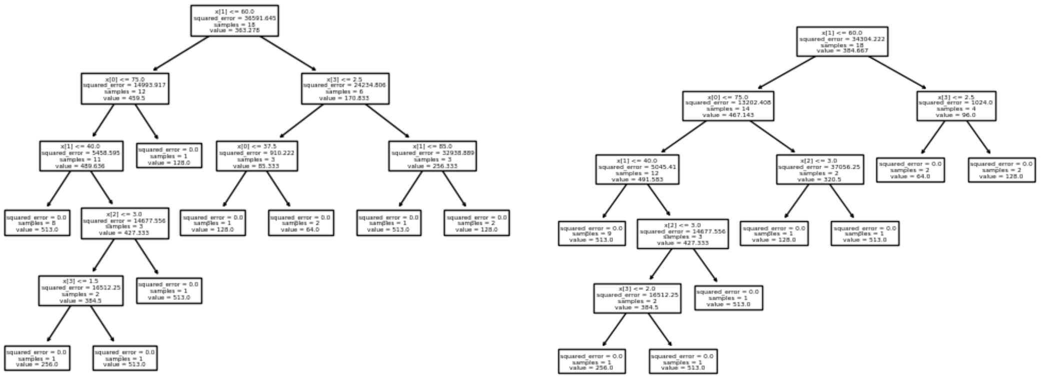

Figure S 11. Different tree structures for MIC USA300 regression decision tree models. Left: tree fitted in run 2. Right: tree fitted in run 4. X[0] = DP, x[1] = CatMonPerc, x[2] = PolyConf, and x[3] = CatMonType.

For classification decision trees, very simple trees were fitted. As most of the datapoints belonged to class 0 for MIC outputs, small, simple trees were effective at fitting this majority data class, even though they were not good with class 1. Tree structures, despite being simple, varied between

different runs for all output variables, even MIC USA300 and MIC Newman. Figure S 12 shows two tree structures for MIC Newman as examples. As seen, the trees are very simple, and some features are not even present. This explains why feature importance for some features in this model is zero (see Table S 4), as feature importance is, very basically, related to the number of times a node in a tree splits over a given feature.

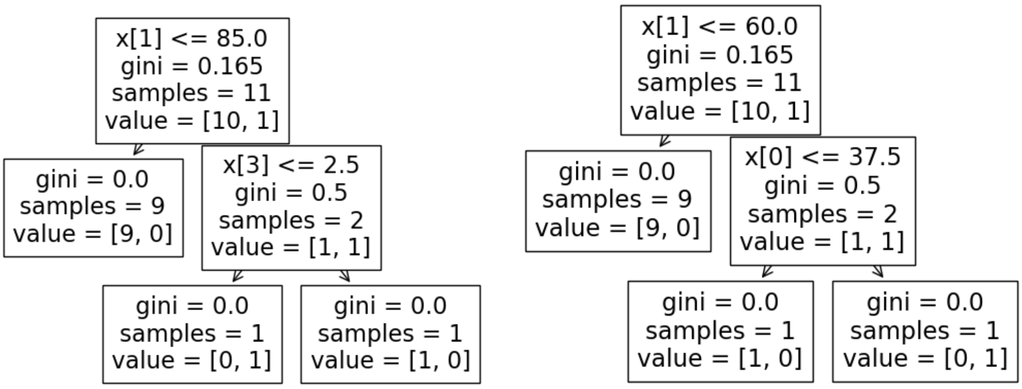

Figure S 12. Different tree structures for MIC Newman classification decision tree models. Left: tree fitted in run 2. Right: tree fitted in run 4. X[0] = DP, x[1] = CatMonPerc, x[2] = PolyConf, and x[3] = CatMonType.

While for both classification and regression decision tree models a few tree structures where similar (and less often identical), many different tree structures were obtained for distinct runs. This could suggest that there are different ways of fitting the data, but from a pragmatic and reliability point of view, models where similar levels of feature importance with small deviation between runs are achieved regardless of how the dataset is randomly selected, are preferable. Decision tree models were thus discarded from further analysis as they did exhibit reproducible feature importance distributions and consistent tree structures.

For gradient boosting, tree structures were analysed for regression models. It was found that the first tree in the sequence had similar node structure and feature selection across different runs, and that the last tree was identical or very similar across different runs.

Figure S 13 shows two initial trees for the MIC PA14 GB regression model. It is worth noting that GB fits trees on the error between actual and predicted output values, and so the value shown in the nodes is that of the average error for all datapoints passing through that node (so the closer to 0 that value is, the better the fit). Figure S 14 then shows the final tree structures for the same runs, and they are identical.

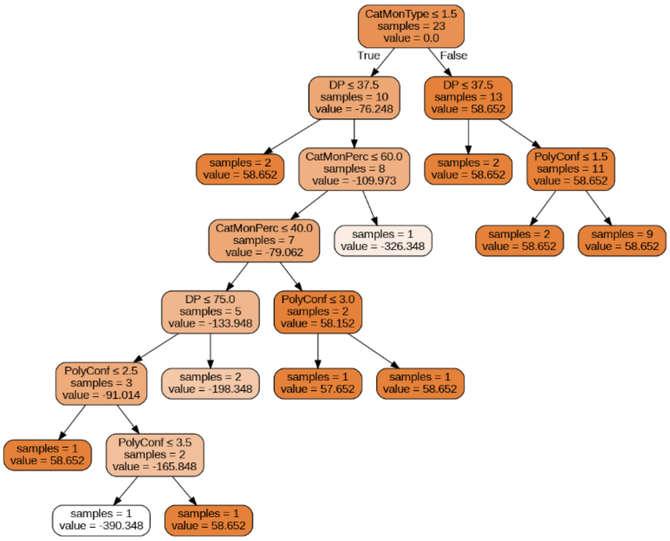

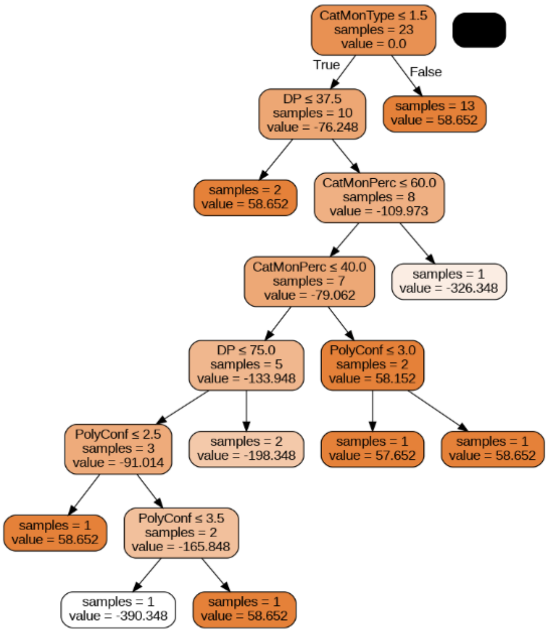

Figure S 13. Initial tree structures for run 1 (up) and run 2 (bottom) of the MIC PA14 GB regression model.

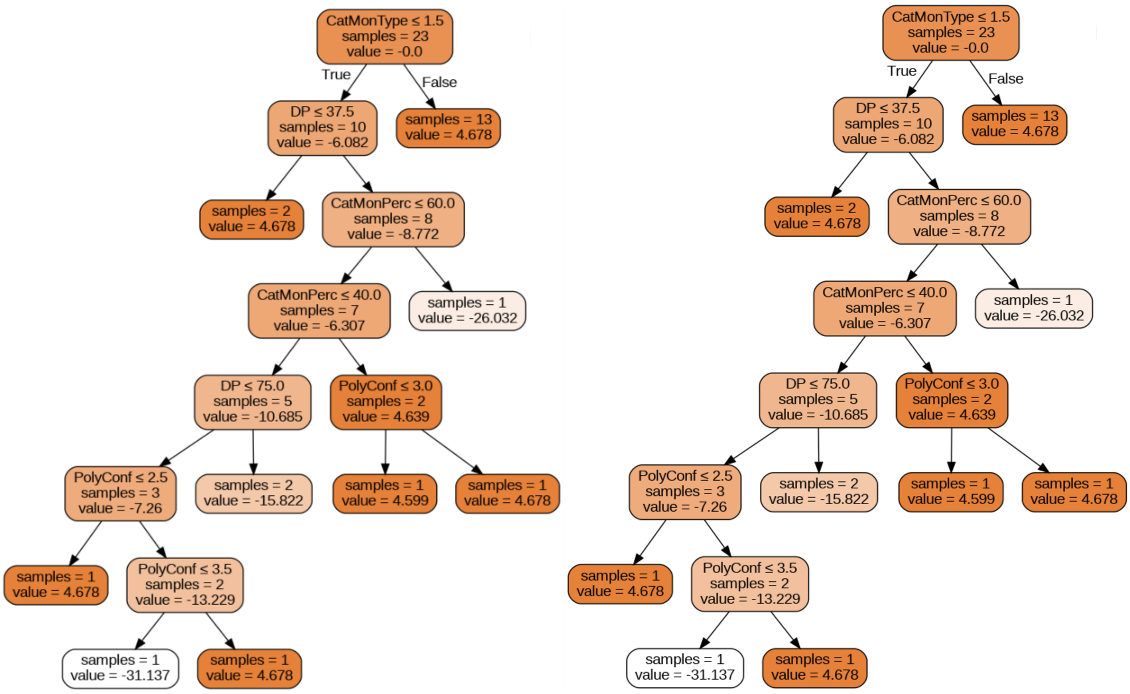

Figure S 14. Final tree structures for run 1 (up) and run 2 (bottom) of the MIC PA14 GB regression model.

This was observed for all output variables. For MIC Newman, for example, the final tree structure was the same fir runs 1 through 4, and for run 5, there were only two more, distinct node splits at layer #5 (Figure S 15).

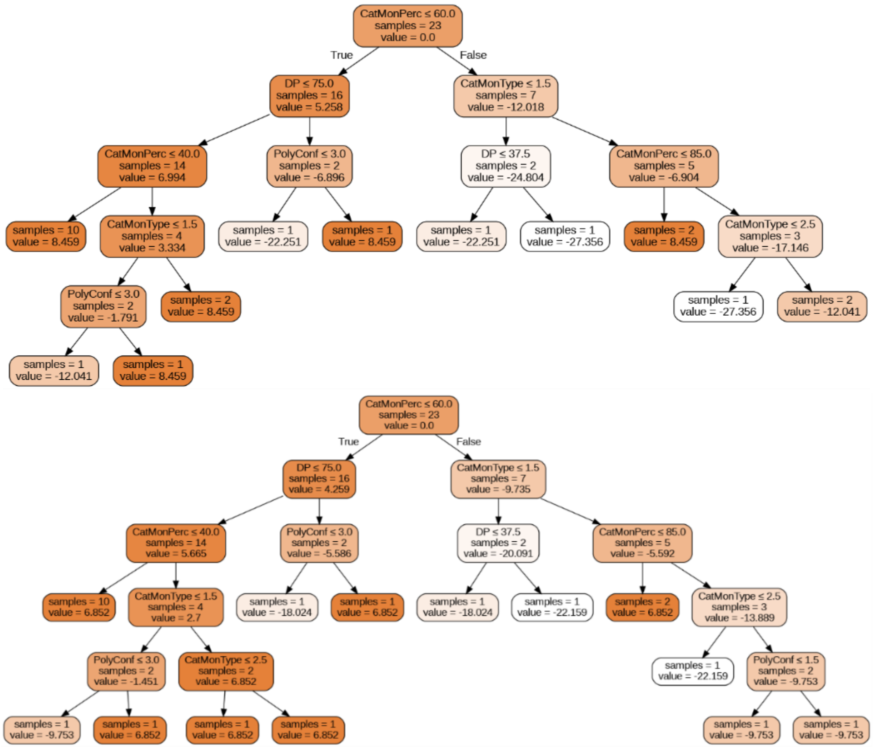

Figure S 15. Last trees for runs 1-4 (top) and run 5 (bottom) for MIC Newman GB regression models.

As tree structures were very consistent for GB regression models, feature importance for these models had very small (rounding to zero) variation. The small additional nodes at deeper layers of the tree do not have as much impact on feature importance as layers higher up, because as shown

by the equation for node importance, these are weighed by the fraction of total samples that pass through the node. The lower the layer the node is in, the smaller the fraction of samples passing through it, and the smaller the importance of that node.

Classification GB models presented high variability of feature importance, and so they were discarded from the model selection on that basis. For RF models, in a similar way, classification models were discarded as they were not as reproducible as regression models. While for MIC outputs only a few datapoints were available for class 1, which might point to the difficulty in classification models reproducing good fits coming from the nature of the dataset itself, for agglutination there was a good split between classes. However, even for agglutination classification models were not reproducible for decision tree and GB (see Table S 4 for average standard error of feature importance of 51% and 47% for decision tree and GB, respectively). For RF, the average standard error of feature importance for agglutination classification model was 17%, considerably lower than decision tree and GB, but still almost three time as high as the average standard error for agglutination regression RF model (6%) Estimating missing agglutination values with RF and GB regression models

Table S 17 shows the estimated agglutination values for the missing datapoints, using both the RF and Gb regression models. The average percent difference between the models was 40%. The difference was lower for most datapoints, ranging between 0-21%. For two datapoints 6 and 22, RF estimated values roughly double those of GB. Otherwise, both models provided similar estimates for missing experimental data.

Table S 17. Estimated agglutination values and percent

					Estimated agglutination (µg/ml)		
Data point #	DP	CatMonPerc	PolyConf	CatMonType	RF regression	GB regression	% Difference
5	50	100	1	2	382.6	432.4	12
6	50	100	1	3	154.7	64	83
10	50	70	2	3	177.7	144.6	21
14	50	50	2	3	254.8	253.7	0
22	100	30	2	1	102.8	32.1	105
23	100	30	4	1	157.5	128	21

Comparing feature importance and average SHAP values for RF and GB regression models

Table S 18 and Table S 19 list the values for average SHAP value and feature importance for RF and GB regression models, as depicted in Figure 4 in the main text.

Table S 18. Average SHAP and feature importance values for RF regression model

		DP	CatMonPerc	PolyConf	CatMonType
**MIC-PA14**	*Feature importance*	0.38	0.16	0.22	0.24
	Average SHAP value	0.18	0.14	0.18	0.50
**MIC-LESB58**	*Feature importance*	0.33	0.25	0.10	0.32
	Average SHAP value	0.23	0.29	0.02	0.46
**MIC-USA300**	*Feature importance*	0.12	0.60	0.13	0.15
	Average SHAP value	0.14	0.56	0.09	0.22
**MIC-Newman**	*Feature importance*	0.12	0.55	0.14	0.19
	Average SHAP value	0.11	0.54	0.10	0.25
**C_H**	*Feature importance*	0.14	0.26	0.26	0.35
	Average SHAP value	0.23	0.17	0.23	0.37

Table S 19. Average SHAP and feature importance values for GB regression model

		DP	CatMonPerc	PolyConf	CatMonType
**MIC PA14**	*Feature importance*	0.16	0.26	0.33	0.25
	Average SHAP value	0.18	0.22	0.16	0.45
**MIC LESB58**	*Feature importance*	0.36	0.37	0.10	0.17
	Average SHAP value	0.28	0.28	0.09	0.35
**MIC USA300**	*Feature importance*	0.07	0.52	0.14	0.26
	Average SHAP value	0.14	0.51	0.12	0.23
**MIC Newman**	*Feature importance*	0.09	0.57	0.17	0.18
	Average SHAP value	0.14	0.48	0.13	0.25
**Agglutination**	*Feature importance*	0.18	0.22	0.20	0.40
	Average SHAP value	0.14	0.25	0.25	0.37

Preliminary multi-objective optimisation: particle swarm optimisation (PSO) and pareto-front analysis

Table S 20 lists some polymer designs obtained by PSO and pareto front analysis when performing multi-objective optimisation on all modelled outputs (MIC PA14, MIC LESB58, MIC USA300, MIC Newman and agglutination). The table also lists the corresponding predicted values for each output variable.

PSO was implemented in Python with the pyswarms module, using default hyperparameters.[1] The objective function was set with equal weight given to each output model, minimising all 4 MIC outputs and minimising the inverse of agglutination concentration (maximising agglutination concentration). The models generated in our work were used in the objective function. Pareto front analysis was implemented with the paretoset package[2]. Points at the pareto front were considered as closest to optimal, though some were excluded due to physical impossibilities (i.e., 0% cationic monomer percent).

Table S 20. Polymer design "hits" from multi-objective optimisation and corresponding predicted output values

Polymer design features				Predicted output values				
DP	CatMonPerc	PolyConf	CatMonType	MIC PA14	MIC LESB58	MIC USA300	MIC Newman	Agglutination
49	69	3	1	151	64	87	88	301
76	41	3	1	507	128	147	148	533
38	61	3	1	151	64	87	88	301
76	86	3	2	508	128	87	88	516

Of the polymer design "hits" listed in Table S 20, two achieve the best compromise. These are designs that feature percentage of cationic monomer higher than 60% with triblock polymer conformation and AEAM monomer. This design achieves low MICs for LESB58, USA300 and Newman strains (predicted 64-88 μg/mL), relatively low MIC for PA14 (151 μg/mL), and relatively high agglutination concentration (300 μg/mL). Another design features even higher percentages of cationic monomer also with a triblock copolymer but with DMAEMA monomer, achieving a good compromise between MIC USA300 and Newman and Agglutination (around 88 μg/mL for the MICs and 516 μg/mL for agglutination). Moderate compromise is achieved with this second design for MIC LESB58 (predicted 128 μg/mL), but poor compromise with MIC PA14 (508 μg/mL). Caution should be exercised as these designs have not been validated experimentally and the optimisation strategy has not been optimised itself.

[1] Tisimst and Castillohair. (n.d.). *pyswarm* [GitHub repository]. GitHub. https://github.com/tisimst/pyswarm

[2] Tommyod. (n.d.). *paretoset* [PyPi.org repository]. Pypi.org. https://pypi.org/project/paretoset/
